# Yellow Fever Outbreaks Are Not Associated With Long‐Term Negative Human Perceptions of Wild Primates in Brazil

**DOI:** 10.1002/ajp.70219

**Published:** 2026-10-21

**Authors:** Cristina Caparrós‐Vallcorba, Filipe Vieira Santos de Abreu, Danilo Simonini‐Teixeira, Waldney Pereira‐Martins, Antonio Victor Veloso‐Ramos, Sofía Bernal‐Valle, Mariana Martins‐Carvalho, Sandy Micaele Aquino‐Teixeira, Naiane Jardim, Thayná Senario, Hermes P. Almeida, Claudio Azat, Julio A. Benavides

**Affiliations:** ^1^ One Health Institute y Doctorado en Medicina de la Conservación, Facultad de Ciencias de la Vida Universidad Andrés Bello Santiago Chile; ^2^ Insect Behavior Laboratory Instituto Federal do Norte de Minas Gerais Salinas Brazil; ^3^ Department of Agricultural and Environmental Sciences Universidade Estadual de Santa Cruz Ilhéus Brazil; ^4^ Departamento de Biologia Geral, Centro de Ciências Biológicas e da Saúde, Universidade Estadual de Montes Claros Universitário Professor Darcy Ribeiro Montes Claros Minas Gerais Brazil; ^5^ Programa de Pós‑Graduação em Biodiversidade e Uso dos Recursos Naturais Universidade Estadual de Montes Claros Montes Claros Minas Gerais Brazil; ^6^ Centro Nacional de Innovaciones Biotecnológicas (CENIBiot) CeNAT, CONARE San José Costa Rica; ^7^ Health Department at Municipal Government of Ubaí Brazil; ^8^ MIVEGEC, IRD, CNRS Université de Montpellier Montpellier France

**Keywords:** disease risk perception, ethnoprimatology, human dimensions, human‐wildlife coexistence, yellow fever

## Abstract

Human perceptions of wildlife are fundamental to guiding effective strategies for conserving biodiversity and public health. Zoonotic diseases affecting non‐human primates, such as yellow fever (YF), may trigger negative human behaviors toward these species. We conducted 474 semi‐structured interviews across seven municipalities in Minas Gerais (*n* = 324 respondents) and Bahia (*n* = 150), Brazil, and used mixed‐effects models to evaluate whether human perceptions of marmosets (*Callithrix* sp.) and howler monkeys (*Alouatta* sp.) differed according to previous YF outbreaks. YF occurrence was not consistently associated with negative perceptions of either species or with perceived disease risk from primates. Instead, more frequent sightings, higher education level, and being male were associated with more positive perceptions of howler monkeys, while marmoset perceptions remained relatively constant. Education was also associated with greater knowledge of disease transmission and lower perceived disease risk from primates, whereas women reported greater risk perception. Because education level differed among municipalities, we further examined the robustness of its association with howler monkey perceptions using sensitivity analyses, which yielded qualitatively similar results. Together, these findings suggest that socio‐demographic and experiential factors may be more strongly associated with human perceptions of non‐human primates than previous YF occurrence.

## Summary

- Previous yellow fever outbreaks were not associated with long‐term negative perceptions of non‐human primates in Brazil.
- Perceptions of howler monkeys were associated with education level, gender, and sighting frequency, whereas no significant predictors were identified for marmoset perceptions.
- Long‐term human perceptions of non‐human primates were more closely associated with socio‐demographic and experiential factors than with previous yellow fever outbreaks.

## Graphical abstract

Previous Yellow Fever outbreaks were not associated with long‐term negative human perceptions of non‐human primates in Brazil. Instead, education level, gender and sighting frequencies shaped perceptions of howler monkeys, while no significant predictors were identified for marmoset perceptions.

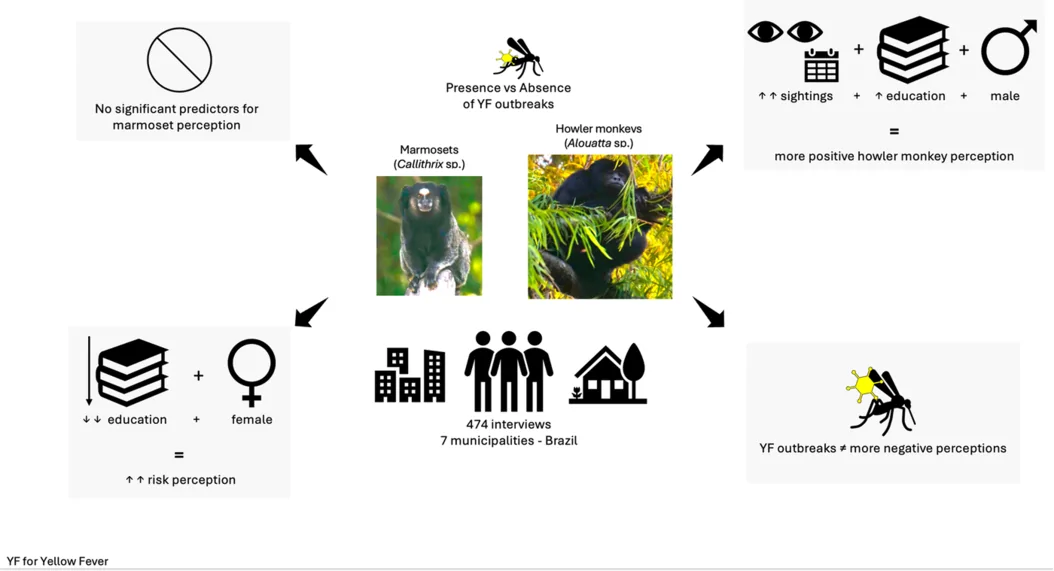

## 1 Introduction

Human‐wildlife coexistence is strongly influenced by how humans perceive and evaluate wildlife, including the perceived benefits, risks and costs associated with coexistence, whether tangible (e.g., economic) or intangible (e.g., emotional responses) (Kansky et al. 2016). In this context, perceptions broadly refer to how individuals interpret and evaluate wildlife and coexistence situations, whereas attitudes represent favorable or unfavorable evaluations towards a given species. Positive attitudes towards wildlife may facilitate tolerance and support for conservation actions, although attitudes do not necessarily translate directly into behaviors, which are also shaped by contextual and social factors (Kansky et al. 2016; IUCN 2023). For instance, favorable views of amphibians by tourists visiting the Pyrenean Mountains resulted in greater support for conservation measures such as restricting public access to sensitive natural areas (Loyau and Schmeller 2017). Even controversial species associated with negative impacts may elicit positive attitudes. For example, snow leopards (*Panthera uncia*) in China are often respected or revered despite causing livestock losses (Hacker et al. 2021), while invasive gray squirrels (*Sciurus carolinensis*) are affectionately regarded by the British public despite displacing native fauna (Dunn et al. 2018). Positive attitudes towards wildlife can, however, be influenced by negative experiences, triggering fear‐driven responses such as the mass culling of Egyptian fruit bats (*Rousettus aegyptiacus*) in Uganda following zoonotic Marburg virus disease outbreaks (Amman et al. 2014), or the illegal killing of large carnivores (such as bears, wolves and lynxes) in response to increasing conflict after their recent recolonization in Europe (Pohja‐Mykrä and Kurki 2014). Therefore, it is crucial to understand the psychological and sociocultural drivers that shape positive and negative attitudes of humans towards wildlife.

Non‐human primates (NHPs) occupy a unique position in human‐wildlife coexistence because of their phylogenetic proximity, shared biological features and frequent spatial overlap with human populations. As a result, they engage in a wide range of context‐dependent relationships with people (Caparrós‐Vallcorba et al. 2026), from cultural reverence to antagonism, with an estimated 30% of all NHP species occurring near anthropogenic settlements (Galán‐Acedo et al. 2019). Examples include the veneration and feeding of Hanuman langurs (*Semnopithecus entellus*) in Indian urban temples as embodiments of the Hindu deity Hanuman, while also serving as tourist attractions (Vijayaraghavan et al. 2021). In Ghana, ursine colobus (*Colobus vellerosus*) has traditionally been protected by taboos that restrict their killing, despite several NHP species being hunted for bushmeat in other African regions (Saj et al. 2006). In Latin America, some indigenous Popoluca communities in Mexico attribute predictive power to howler monkeys' vocalizations for forecasting weather changes, while also sustaining myths about them kidnaping or sexually abusing women (Pinto‐Marroquin and Serio‐Silva 2020). In Brazilian cities, urban residents often show positive attitudes toward marmosets (*Callithrix* sp.), capuchins (*Sapajus* sp.) and howler monkeys (*Alouatta* sp.), which are frequently provisioned and tolerated in public spaces (Leite et al. 2011; Suzin et al. 2017; Alesci et al. 2022). However, negative perceptions of NHPs are also common, driven by both tangible costs such as the economic losses resulting from crop‐foraging (Hill 2000), and intangible ones including fear of aggression (Kazaba et al. 2020) or zoonotic disease transmission (Bicca‐Marques and de Freitas 2010). Since perceptions and attitudes can influence wildlife tolerance, negative evaluations of NHPs may contribute to antagonistic behaviors toward these species (Kansky et al. 2016). Yet, the drivers shaping these negative attitudes and risk perceptions remain poorly understood, limiting our ability to mitigate them and promote a positive human‐NHPs coexistence.

Zoonotic disease transmission is likely one of the most negative outcomes of human‐NHP interactions. NHPs are affected by several diseases causing major public health threats to humans including Zika virus disease, Mpox, rabies and yellow fever (YF), often without a clear understanding of their role in the infectious agent cycle (Kotait et al. 2018; Terzian et al. 2018; Abreu et al. 2020; Besombes et al. 2023). In Brazil, recent YF outbreaks have posed severe risks to unvaccinated human populations, with the 2016‐2018 epidemic being the most significant in the country in the last 70 years (Silva et al. 2020). Due to their high susceptibility to YF, platyrrhines also experienced high mortality during these outbreaks. In particular, howler monkeys (*Alouatta* sp.) populations have been frequently decimated locally during YF outbreaks that can also affect humans (Abreu et al. 2020). But beyond their epidemiological vulnerability, howler monkeys also suffered from human retaliation during outbreaks, amid fears that they transmit YF to humans. For example, fear and misinformation led to the persecution and killing of howler monkeys by residents during the 2008‐2009 outbreak in Rio Grande do Sul, southern Brazil, reducing already impacted populations by up to an additional 40% (Bicca‐Marques and de Freitas 2010). Over the past decade, reports of human aggression towards NHPs during YF outbreaks have persisted, particularly targeting howler monkeys, and have been further reinforced by concerns over other emerging diseases such as Mpox (Martín 2018; Passarinho 2018; Lenthang 2022). Despite the severity of these incidents, no study has yet assessed the long‐term drivers of human attitudes and risk perceptions towards NHPs in YF outbreak‐affected areas, or whether these negative attitudes persist after the end of these outbreaks.

Zoonotic diseases are not the sole drivers shaping human perceptions and attitudes of NHPs in Brazil. The common marmoset (*Callithrix jacchus*) is the only primate species currently recognized as a reservoir of a human rabies variant (Kotait et al. 2018), while recent reports have also detected the virus in the black‐tufted marmosets (*Callithrix penicillata*) (Souza Júnior et al. 2024). In addition, *Callithrix* species are susceptible to YF (Abreu et al. 2020). Increasing reports of rabies in the common marmosets (*Callithrix jacchus*) (Benavides et al. 2022) and cases of human rabies associated to marmoset bites (Bento 2025) underscore their relevance as a public health concern. Yet, this species seems to benefit from generally positive attitudes among humans (Leite et al. 2011; de Albuquerque and de Oliveira 2014). Thus, more favorable attitudes towards marmosets compared to howler monkeys suggests that species‐specific perceived risks and benefits may shape human attitudes and tolerance differently across NHP species.

Here, we use semi‐structured interviews administered to local communities sharing habitat with marmosets (*Callithrix* spp.) and howler monkeys (*Alouatta* spp.) to study the correlation of human perceptions with several drivers including perceived zoonotic risk. Specifically, we aim to (i) test whether human perceptions towards NHPs are correlated with the occurrence of prior YF outbreaks (Bicca‐Marques and de Freitas 2010), species sighting frequency (Kansky et al. 2016), urbanization level, and socio‐demographic characteristics such as education level, gender, and age (Manfredo et al. 2020; IUCN 2023); and (ii) assess if perceived risk of humans towards howler monkeys and marmosets regarding YF, COVID‐19, Mpox, and rabies, vary according to the YF outbreak area.

## 2 Materials and Methods

### 2.1 Ethical Considerations

The study protocol was evaluated and approved by the Bioethics Committee of Life Sciences Faculty, Universidad Andrés Bello, Chile (021/2022), and by the Ethics Committee in Research of Universidade Estadual de Santa Cruz, Brazil (62117822.0.0000.5526).

### 2.2 Study Area and Municipality Selection

The study was conducted in five municipalities of Minas Gerais (MG) and two municipalities of Bahia (BA), Brazil, between August and November 2023 (Figure 1). We designed the study to include at least one municipality in each of the 4 combinations: urban YF‐positive, rural YF‐positive, urban YF‐negative, and rural YF‐negative. Urban and rural areas within each selected municipality were defined according to geospatial data obtained from the Brazilian Institute of Geography and Statistics (IBGE 2024) (see Supporting Information S1: Table S1 for detailed definitions). We began by selecting two neighboring states: one with previous YF outbreaks in humans (MG) and the nearest state without autochthonous human outbreaks reported (BA) (Goes de Jesus et al. 2020). In MG, the 2016–2018 YF outbreak caused substantial morbidity and mortality in both humans and NHPs (Sacchetto et al. 2020; Silva et al. 2020). More recent official reports documented a limited number of YF epizooties in 2022 (10 epizootics in MG, 7 in Brasilia de Minas and 1 in Ubai) (Ministério da Saúde do Brasil 2023), while municipal communications also reported suspected dead NHPs during the study period (Prefeitura de Brasília de Minas 2023). In contrast, BA reported no autochthonous cases of YF in humans from 2000 until the study date, although it reported cases in NHPs in 28 municipalities in 2017 (Goes de Jesus et al. 2020; Ministério da Saúde do Brasil 2023; PAHO/OMS 2024). Then, we identified municipalities within each state with confirmed occurrence of marmoset (*Callithrix* sp.) and howler monkey (*Alouatta* sp.) species, using data obtained from SISS‐Geo (sissgeo.lncc.br/) and Culot et al. (2019). These municipalities were located within three different biomes (Caatinga, Cerrado and Mata Atlântica; IBGE).

**Figure 1:**
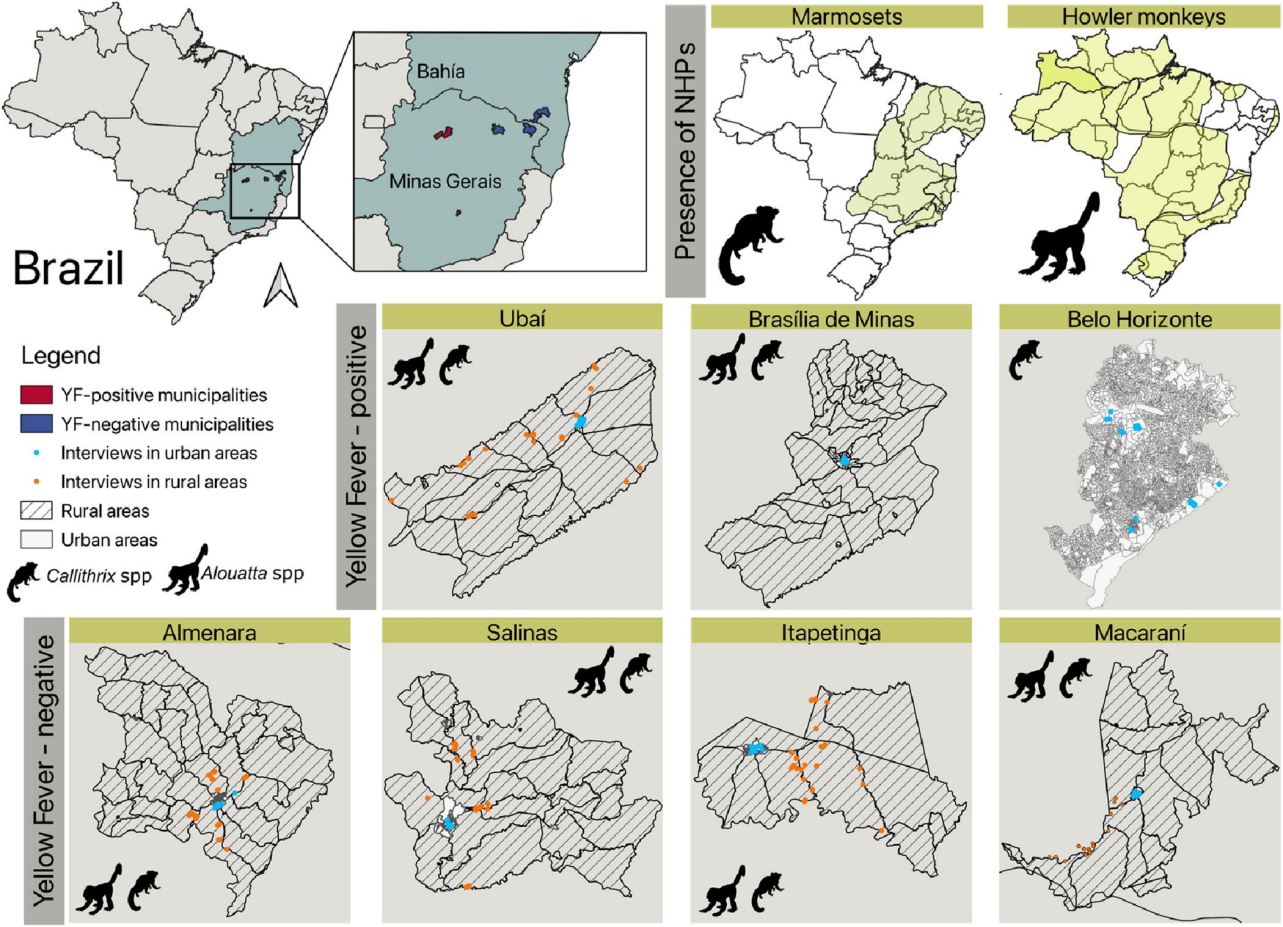
Geographical location of study municipalities, sampled urban and rural areas, and distribution of *Callithrix* spp. and *Alouatta* spp. in Brazil. Both genera were present in all study municipalities, although *Alouatta* spp. occurred mainly in forested regions surrounding Belo Horizonte (IUCN 2024). IUCN. 2024. The IUCN Red List of Threatened Species.

The identified municipalities were then categorized as previously “YF‐positive” or “YF‐negative” based on public epidemiological data of YF outbreaks from 1994 to 2023. YF‐positive municipalities had confirmed outbreaks of YF in humans, NHPs, or both, during the 5 years preceding the study (see Supporting Information S1: Table S2; Ministério da Saúde do Brasil 2023). Brasilia de Minas also reported suspected cases in NHPs in 2023, without official confirmation from the health ministry (Prefeitura Brasília de Minas 2023). According to our logistical possibilities, three YF‐positive municipalities were selected: two with the most recent confirmed NHP YF cases–Ubaí (*n* = 85 respondents, urban and rural) and Brasília de Minas (*n* = 36, urban)–and the largest urban center and state capital, Belo Horizonte (*n* = 54, urban), which reported both human and NHP YF cases (Ministério da Saúde do Brasil 2023). The selected YF‐negative municipalities had not reported YF outbreaks since 1999, were located close to the YF‐positive municipalities and the BA border and had recent reports of NHP presence (marmosets and howler monkeys) by members of our research team. These included Salinas (*n* = 73) and Almenara (*n* = 76, both urban and rural) in MG, along with the nearest municipalities in BA, Macaraní (*n* = 75) and Itapetinga (*n* = 75, both urban and rural) (Figure 1). In Belo Horizonte, documented howler monkey occurrences were associated with surrounding forested regions rather than the surveyed urban area.

In total, we sampled three YF‐positive urban areas (*n* = 131 interviewees), one YF‐positive rural area (*n* = 44), four YF‐negative urban areas (*n* = 146), and four YF‐negative rural areas (*n* = 153) (Table 1, see Supporting Information S1: Table S3).

**Table 1:** Summary of respondent demographics.

	YF‐positive	YF‐negative
Men	71 (41%)	172 (58%)
Women	104 (59%)	127 (42%)
Mean age (years old)	48	49
Urban	131 (75%)	146 (49%)
Rural	44 (25%)	153 (51%)
No secondary education	73 (42%)	217 (73%)
Secondary education	41 (23%)	55 (18%)
More than secondary education	61 (35%)	27 (9%)
Total	175	299

### 2.3 Data Collection

Semi‐structured interviews were conducted with residents encountered in or around their homes, who were invited to participate voluntarily. In urban areas, participants were recruited through random sampling from neighborhoods located between 0 and 1.5 km from green areas (i.e., forest fragments edges) identified using Google Maps, where most human‐NHP interactions occur (Sacchetto et al. 2020). Interviewers remained in each selected neighborhood until they had approached all the households and residents who were present. In rural areas, public health agents from each municipality assisted in locating and approaching residents within zones identified as rural using IBGE data. All rural residents who were present in their homes were invited to participate until the target sample size was reached.

Inclusion criteria consisted of being over 18 years of age, residing in the study area, and providing written informed consent. All but two respondents had lived in the area for at least 1 year (the two exceptions had resided there for 5 and 9 months, respectively). Each interview lasted between 30 and 50 min and was conducted in Portuguese using the open‐source KoboToolbox software on mobile devices (www.kobotoolbox.org/).

### 2.4 Sample Size Estimation

To quantitatively compare NHP perception rates between regions with and without YF outbreaks in Brazil, we focused on howler monkeys, as they are particularly affected by human persecution driven by YF concerns (Bicca‐Marques and de Freitas 2010). Due to a lack of prior studies evaluating human perception of howler monkeys in Brazil, we conservatively assumed a baseline positive‐perception rate of 50% in YF‐negative areas, which maximizes variance in proportion‐based sample size calculations. We then aimed to detect at least a 20% reduction in positive‐perception rates in areas with YF outbreaks, with a desired power of 90% and a confidence level of 99%. This required a sample size of 177 interviews per group (YF‐positive, YF‐negative), resulting in a total sample size of 354 individuals. During the fieldwork phase, more advantageous conditions in YF‐negative areas (i.e., increased help from public health administrations) allowed us to increase the number of interviews in YF‐negative areas to 299, whereas 175 interviews were conducted in YF‐positive areas.

### 2.5 Semi‐Structured Interviews

Questionnaires with closed and open‐ended questions were administered, enabling participants to share experiences and feelings beyond the pre‐defined options. As part of a broader project, the interview comprised five dimensions: (1) Knowledge and contact with NHPs (21 items); (2) Perceptions of marmosets and howler monkeys (6 items per species); (3) Acceptability of contact reduction strategies (7 items); (4) Disease risk perception (14 items), and (5) Socio‐demographic characteristics (5 items) (see Supporting Information S2).

Marmoset and howler monkey identification was evaluated using 12 images of distinct species of wildlife (see Supporting Information S2: Figure S1). After the image‐identification task, respondents were shown the correct images of each species before continuing with the interview, in order to standardize species recognition during subsequent perception questions. Perceptions of marmosets and howler monkeys were measured using six questions where respondents rated their level of agreement to a given statement on a semantic differential scale ranging from 1 to 5 (e.g., happy to sad), with a “no opinion” option (see Supporting Information S1: Table S4 for more detail). Semantic differential scales are commonly used to assess attitudes and perceptions across positive‐negative gradients (Friborg et al. 2006).

Risk perception regarding disease transmission from NHPs was measured through seven questions with the following alternatives: “Yes,” “No” and “Do not know/Do not answer“ (“DK/DA”). These included two questions assessing general knowledge and fear of disease transmission by NHPs, and five assessing knowledge of specific diseases transmission (rabies, COVID‐19, YF, and Mpox).

Prior to data collection, the questionnaire was pilot tested with 10 respondents to improve questions wording and structure. Interviewers (*n* = 9) also received prior training before fieldwork regarding questionnaire structure and application, guidance on avoiding interference with respondents' answers, and standardized procedures for the image‐identification task and subsequent explanation of the focal species.

### 2.6 Statistical Analyses

#### 2.6.1 Drivers Associated With Human Perception of Howler Monkeys and Marmosets

We obtained mean and median values of the “human perception” index of each studied NHP species, and tested their associations with several variables obtained from the questionnaires, by implementing linear mixed‐effects models (LMMs) using the function *lmer* from the *lmerTest* package in R. The response variable was squared prior to analysis because the perception indices showed negatively skewed distributions, and this transformation improved the residual diagnostics of the Gaussian mixed models. Gaussian LMMs were preferred over beta regression because the perception indices could take values at the bounds of the scale (1 and 5), which cannot be directly accommodated by standard beta regression without additional modeling or rescaling. In addition, the Gaussian models on the squared perception indices showed adequate residual diagnostics.

The full model included six categorical variables as fixed effects: (i) YF outbreak occurrence (YF‐positive or YF‐negative municipalities), (ii) urbanization (urban/rural), (iii) sighting frequency for each species, and (iv–vi) sociodemographic variables such as age groups (defined as 18–26, 27–39, 40–52, 53–64, > 64, using Braun 1973), gender, and education level (classified as less than secondary education, secondary education, or more than secondary education). The sighting frequency variable was coded into three categories: high frequency (daily or weekly sightings), medium frequency (monthly or occasional sightings, from once to four times per year), and low frequency (less than once per year, including those who had seen them only once, never, or did not know/declined to answer). Municipality and interviewer ID were included as random factors. We built one model per species (howler monkeys and marmosets).

An automated model selection tool (*dredge* function in R) was applied to select the best‐fitting model, based on Akaike's Information Criterion (AIC), with a ∆AIC threshold ≥ 2 per parameter of the model to retain competing models. We then tested the significance of each fixed effect variable in the selected model using the Wald's test. Model residuals were inspected using the *simulateResiduals* function of the *DHARMa* package. Marginal and conditional R^2^ were calculated for the final LMMs to estimate the variance explained by fixed effects alone and by fixed and random effects combined, respectively (Table 2).

**Table 2:** Output from LMMs on squared perception indices for marmosets and howler monkeys in the AIC‐selected models. Estimate values are reported on the squared perception scale.

Fixed effects		Estimate	SE	df	*p* value
Marmosets					
Intercept		15.09	0.83	11.34	**< 0.001** ***
Howler monkeys					
Intercept		13.78	0.77	16.68	**< 0.001** ***
Education	(Secondary education)	0.67	0.60	423.92	0.2683
	(More than secondary education)	1.93	0.67	284.42	**0.0040** **
Gender	(Female)	−1.59	0.48	422.96	**< 0.001** ***
Sighting frequency	(High frequency)	2.19	1.00	383.99	**0.0289** *
	(Medium frequency)	1.04	0.66	377.89	0.1171

*Note:* Model fit statistics: Marmosets (AIC = 2828.88, marginal *R* ^2^ = 0.000, conditional *R* ^2^ = 0.184); Howler monkeys (AIC = 2600.10, marginal *R* ^2^ = 0.056, conditional *R* ^2^ = 0.181).

Abbreviation: SE = Standard Error.

* *p* < 0.05

** *p* < 0.01

*** *p* < 0.001.

To build the “human perception” index used as the dependent variable in the LMMs, we first assigned a perception scoreto each response ranging from 1 (negative perceptions) to 5 (positive perceptions), with 3 representing neutral responses. “No opinion” responses were treated as missing values. Respondents answering “no opinion” to all six perception questions for a given species were excluded from the corresponding analyses. For respondents with partial missingness, the perception index was calculated as the mean of the available perception items. We then assessed the internal consistency of these six perception scores per species using Cronbach's alpha reliability coefficient (Vaske et al. 2017). The resulting alpha values were considered adequate (0.85 for marmoset perception, 0.88 for howler monkey perception). Therefore, the perception index for each species was estimated as the average score from the six attitude items, similar to Manfredo et al. (2020), and used in the statistical models. Initially, we explored the use of Multiple Correspondence Analysis (MCA) to summarize the six perception items. However, the first two dimensions explained only a small proportion of the total variance (18.1%). Given the high internal consistency of the items for both species, we therefore used a composite perception index based on the average item score instead.

#### 2.6.2 Drivers Associated to Disease Knowledge and Perceived Risk

To evaluate if the knowledge of specific zoonotic diseases was associated to previous YF outbreaks, we compared the frequency of positive answers to each disease‐question between YF‐positive and YF‐negative areas using chi‐squared tests (*p* < 0.05).

We tested the association between disease risk perception regarding NHPs and the same predictors from the human perception model through two questions: (1) “*Do you think that NHPs can transmit diseases?”* and (2) "*Are you afraid that NHPs could transmit diseases to you?*.” Since the response options for these questions were multinomial with three choices (“No,” “Yes,” “DK/DA”), we employed a multinomial logit mixed (MXL) model, fitted using the *mblogit* function from the *mclogit* package in R.

For the first question, the full model included all the variables from the human perception model. For the second question, the full model included the same fixed effects, except for sighting frequency, as its inclusion –even alone– prevented model convergence. To still evaluate the effect of sighting frequency on the second question, a Generalized Linear Mixed Model (GLMM) was also built using a simplified binomial response variable (“Yes” vs. “No”) and excluding ‘DK/DA’ responses, fitted using *glmer* function from *lme4* package. For both questions, we assessed the statistical significance of each variable using a Wald's test on the full model, since AIC‐based automated model selection (*dredge* function) was not compatible with multinomial models. The *DHARMa* package was also incompatible with MXL models. Odds ratios (OR) and 95% confidence intervals were calculated by exponentiating coefficients and their confidence bounds. For the GLMM model, the *dredge* function was applied to select the best‐fitting model, and the residuals were inspected using the *simulateResiduals* function of the *DHARMa* package. All statistical analyses were conducted in R (version 4.2.3; R Core Team 2024).

Variance inflation factors (VIFs) were calculated to assess multicollinearity among fixed‐effect predictors in perception and disease‐risk models.

#### 2.6.3 Sensitivity Analyses

Additional sensitivity analyses were conducted to evaluate the robustness of the main results. For the howler monkey perception model, sensitivity analyses were based on the full additive model including all predefined predictors. To assess the potential influence of educational imbalance between YF‐positive and YF‐negative municipalities, we re‐ran this model after excluding respondents with more than secondary education and fitted an alternative model including an education × YF‐status interaction. Model support was compared using AIC values.

To evaluate alternative classifications of YF exposure, municipalities with reported human YF cases (Belo Horizonte) were distinguished from municipalities with predominantly NHP‐associated YF occurrence (Ubaí and Brasília de Minas), and a three‐level predictor (“no‐YF”, “NHP‐YF”, and “human‐YF”) was used in alternative perception and disease‐risk models.

To assess the influence of species recognition, the full perception models were repeated using only respondents who correctly identified at least one focal species image.

Finally, to examine whether the relationship between gender and howler monkey perceptions was consistent across urban and rural contexts, the same full additive howler monkey perception model was fitted separately for urban and rural respondents.

## 3 Results

### 3.1 Socio‐Demographic Background of the Respondents

We interviewed a total of 474 participants, including 175 individuals (37%) from YF‐positive areas and 299 (63%) from YF‐negative areas, 68% (*n* = 324) from five municipalities of MG, and the remaining 32% (*n* = 150) from two municipalities of BA. We interviewed a higher percentage of urban respondents (58%, *n* = 277) and an even percentage of women (49%). More than half of the respondents (61%) had not completed secondary education, while 20% finished secondary education, and 19% had more than secondary education (Table 1).

### 3.2 NHPs Identification and Sight Frequency

Marmosets were the most frequently sighted species, with 64% of respondents reporting seeing them at least once per week (56% in YF‐negative areas, 78% in YF‐positive areas; Figure 2A). In contrast, only 6% of respondents reported seeing howler monkeys at least once per week (2% in YF‐negative areas, 11% in YF‐positive areas), while 63% reported never seeing howler monkeys (72% in YF‐negative areas, 49% in YF‐positive areas; Figure 2B). Likewise, respondents identified marmoset images more accurately than howler monkey images, with 47% (223/474) correctly identifying both marmoset images (40% in YF‐negative areas, 56% in YF‐positive areas; Figure 2C), compared with 5% (25/474) correctly identifying both howler monkey images (4% in YF‐negative areas, 8% in YF‐positive areas; Figure 2D). When additionally considering respondents who correctly identified at least one image for each species, recognition increased to 95% for marmosets (94% in YF‐negative areas, 96% in YF‐positive areas) and 35% for howler monkeys (26% in YF‐negative areas, 50% in YF‐positive areas).

**Figure 2:**
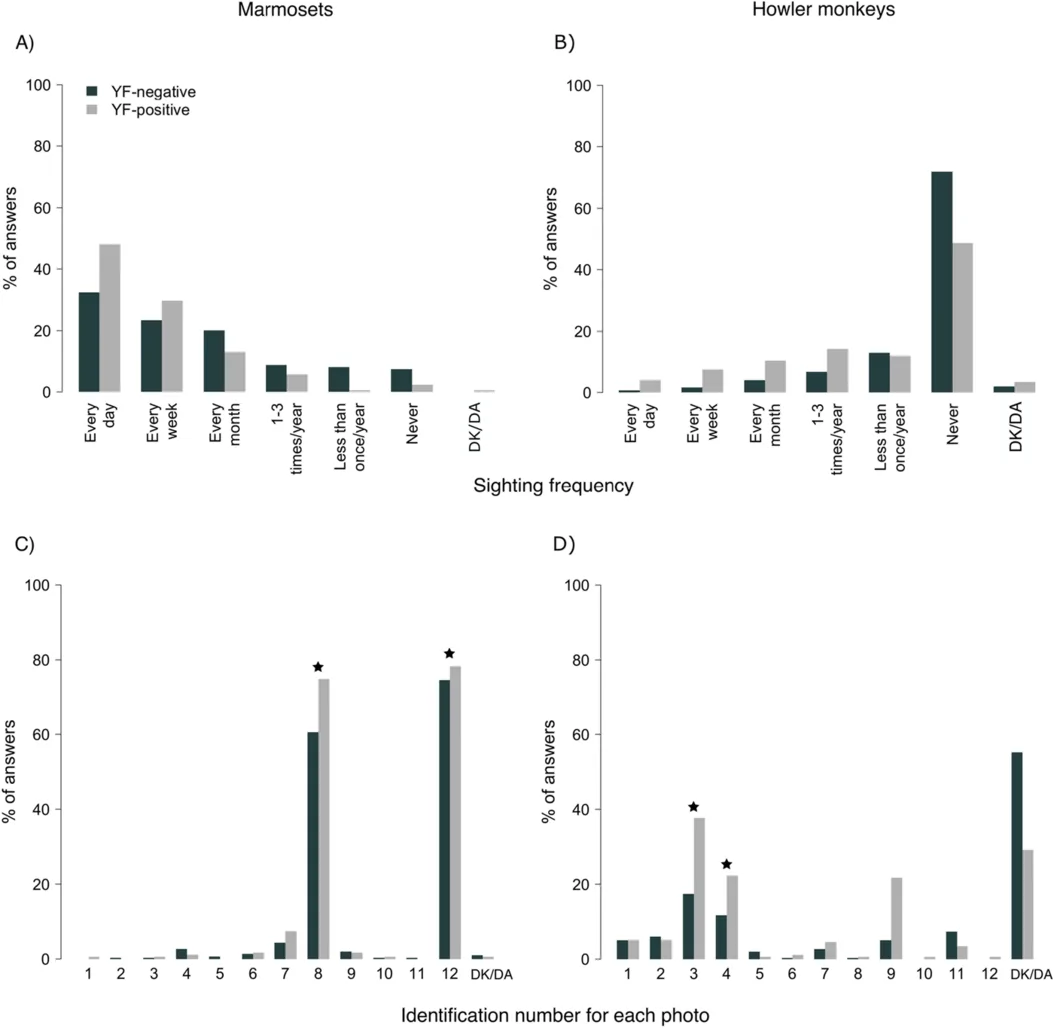
Top graphics: Percentage of responses for the frequency of sightings of marmosets (A) and howler monkeys (B). Each respondent could select only one response for the frequency of sightings of each species. Bottom graphics: Percentage of answers for each image in the photo‐identification of marmosets (C) and howler monkeys (D). Each interviewee could select as many images as they considered. For marmosets, the correct images (★) were the numbers 8 and 12, while for howler monkeys the correct numbers were 3 and 4. Species represented in each photo: (1) Chimpanzee, *Pan troglodytes*; (2) Bald uakari, *Cacajao calvus*; (3) Black and gold howler monkey, *Alouatta caraya*; (4) Brown howler monkey; *Alouatta guariba*; (5) Three‐fingered sloth, *Bradypus variegatus*; (6) Ring‐tailed lemur, *Lemur catta*; (7) Tufted capuchin, *Sapajus apella*; (8) Common marmoset, *Callithrix jacchus*; (9) Wooly spider monkey, *Brachyteles arachnoides*; (10) Big‐eared opossum, *Didelphis aurita*; (11) Orangutan, *Pongo* sp.; (12) Black‐tufted marmoset, *Callithrix penicillata*. See Supplementary Information for more detail.

### 3.3 Factors Associated With Human Perception of Marmosets and Howler Monkeys

Respondents reported a generally positive perception of both marmosets and howler monkeys, with an average perception score of 3.84 out of 5 (median = 4.00) for marmosets and 3.70 for howler monkeys (median = 3.75). Respondents answering “no opinion” to all six perception questions were excluded from the analyses, corresponding to 42 respondents (9%) for howler monkeys and none for marmosets. Among respondents included in the analyses, the number of answered items ranged from 1 to 6 for both species, with a median of six items for marmosets and five items for howler monkeys.

The average human perception index was relatively constant across municipalities for both NHP species (Figure 3). The AIC‐selected LMMs testing human perception with several variables fitted the data appropriately (KS test, dispersion test, and outlier tests all with *p* > 0.05, see Supporting Information S1: Figure S1). Prior YF occurrence was not associated with human perception of either species, as YF occurrence was neither retained in the AIC‐selected model nor significant in the full additive model (*p* > 0.05, see Supporting Information S1: Table S5). For marmosets, none of the tested predictors (i.e., YF occurrence, urbanization, sighting frequency, or socio‐demographic factors) were retained in the final model, indicating uniformly stable perceptions across contexts. In contrast, in the full additive model, human perceptions of howler monkeys were associated with education level, sighting frequency, and gender (Figure 4). These predictors were retained in the AIC‐selected model, with respondents having higher education (more than secondary education) reporting significantly more positive perceptions (LMM, estimate 1.93 on the squared perception scale, *p* < 0.005). Similarly, those who observed howler monkeys frequently (daily or weekly) reported more positive perceptions (LMM, estimate 2.19, *p* < 0.05). Women reported significantly lower perceptions of howler monkeys than men (LMM, estimate −1.59, *p* < 0.001) (Table 2).

**Figure 3:**
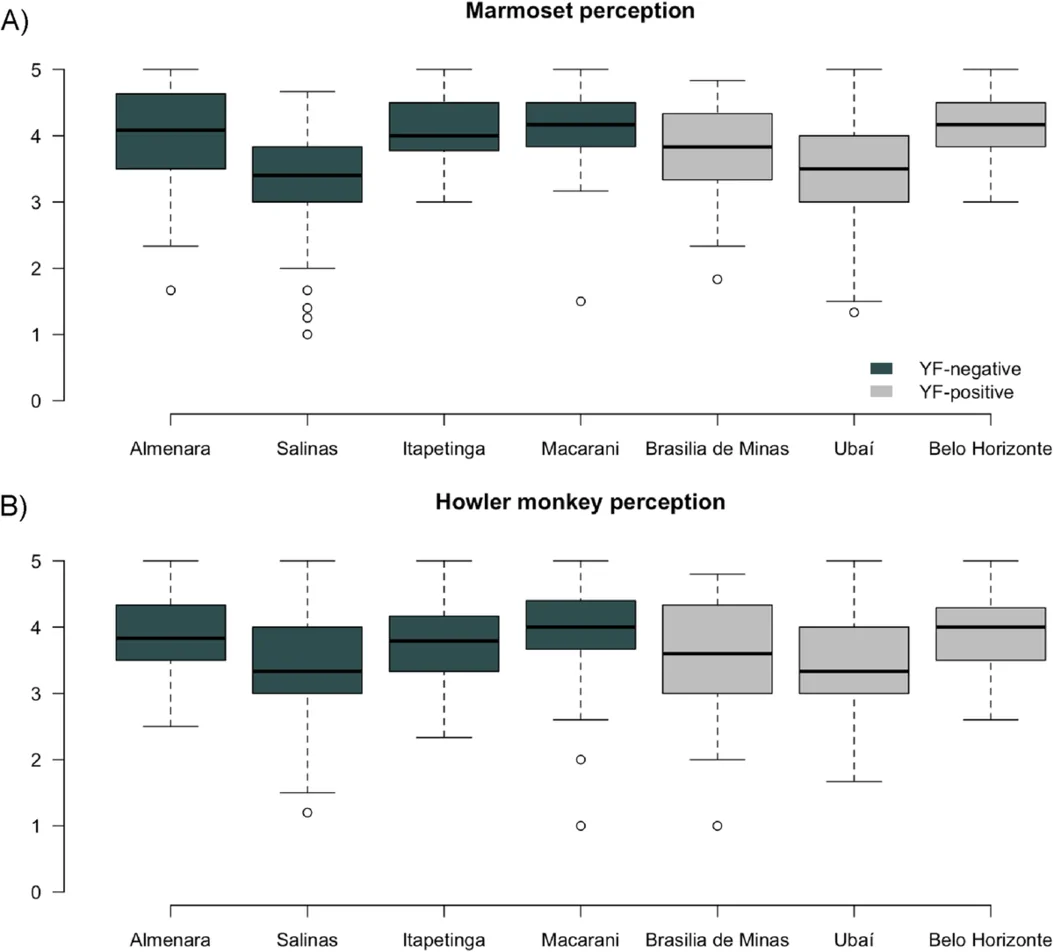
Distribution of average human perception index of marmosets (A) and howler monkeys (B) across municipalities. Boxplots show the median (central line), the interquartile range (IQR; 25th and 75th percentiles), and whiskers extending to the most extreme values within 1.5 × IQR. Values beyond this range are shown as points (outliers). Dark and light gray boxes represent YF‐negative and YF‐positive municipalities, respectively.

**Figure 4:**
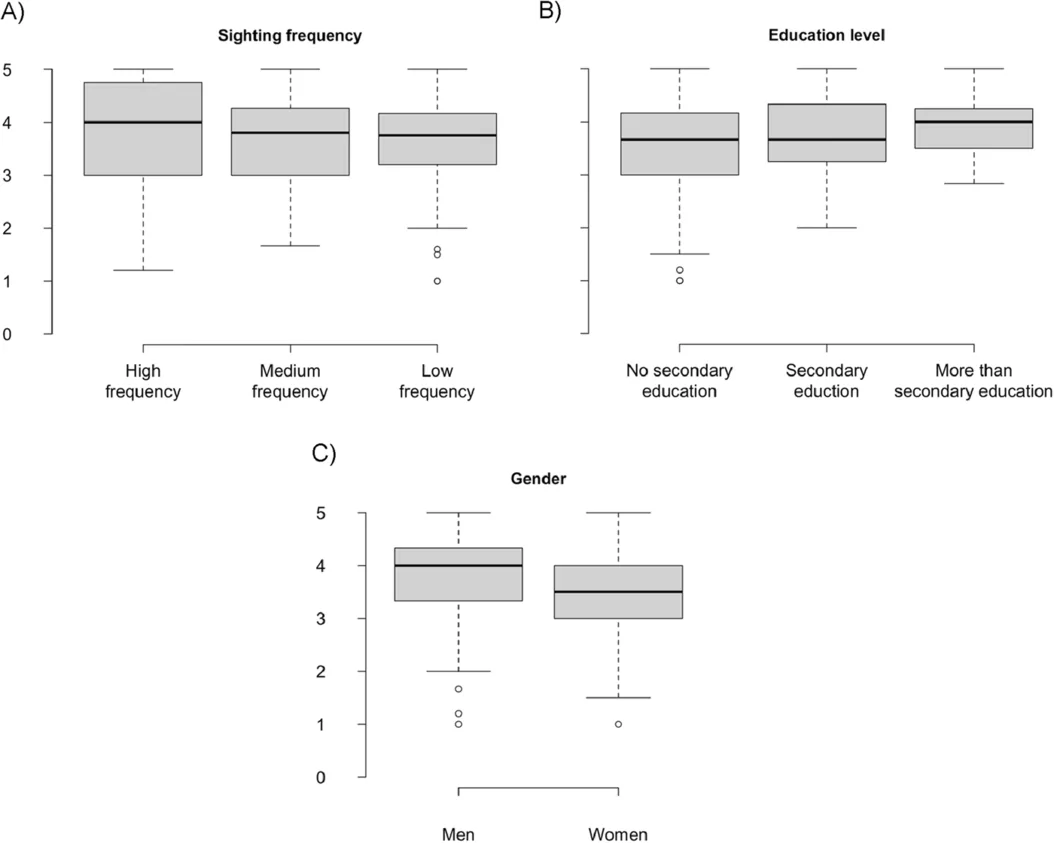
Distribution of human perception index of howler monkeys as a function of the sighting frequency (A), the level of education (B), and the interviewee gender (C).

Sensitivity analyses separating municipalities with human‐YF and NHP‐YF occurrence, as well as accounting for socio‐demographic imbalance between municipalities (i.e., differences in educational composition), yielded qualitatively similar results, with YF‐status remaining non‐significant across models (LMMs, *p* > 0.05) (see Supporting Information S1: Table S6, S7). Similarly, restricting the perception models to respondents who correctly identified at least one focal species image yielded qualitatively similar results for both species (see Supporting Information S1 Table S8). For howler monkeys, education level and gender remained significant predictors, whereas sighting frequency was no longer significant. Finally, when the howler monkey perception model was fitted separately for urban and rural respondents, the association between gender and perception was consistent, with women showing lower perception scores than men in both rural and urban areas (see Supporting Information S1 Table S9).

### 3.4 Factors Associated With Human Perception of Disease Risk Regarding NHPs

Nearly 75% of respondents believed that NHPs could transmit diseases to humans, while 59% expressed fear of such transmission. Beliefs about disease transmission showed no significant differences between YF‐positive and YF‐negative areas (75% vs 73% positive answers; χ^2^ = 2.17, df = 2, *p* > 0.05), nor did fear of disease transmission (58% vs 60% positive answers, Fisher's exact test, *p* > 0.05) (Figure 5).

**Figure 5:**
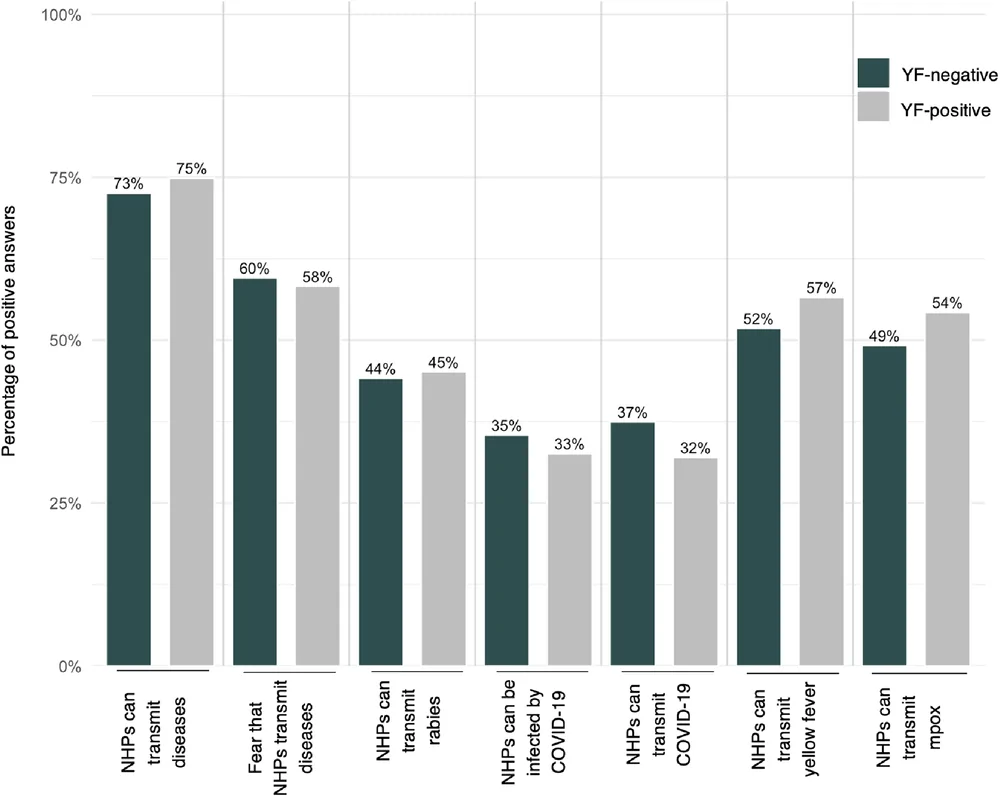
Disease perception risk towards NHPs. Proportion of positive answers (“Yes”) for the seven questions on disease transmission per group (YF‐positive, YF‐negative). From left to right: (1) *Do you believe that NHPs can transmit diseases?*; (2) *Are you afraid that NHPs can transmit diseases to you?*; (3) *Do you believe that rabies could be transmitted by NHPs?*; (4) *Do you believe that NHPs can be infected by COVID‐19?*; (5) *Do you believe that COVID‐19 could be transmitted by NHPs?*; (6) *Do you believe that YF could be transmitted by NHPs?*; (7) *Do you believe that mpox could be transmitted by NHPs?*.

Higher education levels (more than secondary education) were associated with a lower likelihood of reporting uncertainty (i.e., DK/DA) compared to affirming that NHPs could transmit diseases (MXL, OR = 0.36, 95% CI [0.13–0.97], *p* < 0.05) (see Supporting Information S1 Table S10). Similarly, respondents with higher education were more likely to deny fear of such transmission (MXL, OR = 1.81 [1.01–3.23], *p* < 0.05), suggesting lower perceived risk, although both estimates were associated with relatively broad confidence intervals. Women were more likely than men to report fear of disease transmission from NHPs, as indicated by lower odds of denying such fear (MXL, OR = 0.51 [0.35–0.76], *p* < 0.001) (see Supporting Information S1 Table S11). In contrast, neither belief nor fear of disease transmission was associated with YF occurrence or sighting frequency (MXL, *p* > 0.05) (see Supporting Information S1 Table S10, S11). Because the multinomial model including sighting frequency did not converge, this predictor was additionally evaluated using a binomial GLMM contrasting “Yes” and “No” responses. The AIC‐selected final binomial GLMM yielded similar results and retained only gender as a significant factor.

One third of respondents expressed considerable uncertainty about specific disease transmission routes involving primates. For instance, 32% were unsure whether NHPs could transmit rabies, 29% about direct transmission of YF, and 38% about Mpox. Approximately half of the respondents believed that NHPs could transmit YF (54%), Mpox (51%), and rabies (45%), yet no significant differences were observed between municipalities with or without reported YF outbreaks (χ^2^ = 2.35, df = 2, *p* > 0.05 for YF; χ^2^ = 1.28, df = 2, *p* > 0.05 for Mpox; χ^2^ = 1.62, df** = **2, *p* > 0.05 for rabies). When asked to specify which NHP species were involved in the transmission of YF, Mpox or rabies, nearly 90% of respondents either indicated that they did not know, or stated that all NHPs were equally capable of transmitting the disease. Knowledge about potential COVID‐19 transmission between NHPs and humans was more evenly distributed (35% answering positively, 28% negatively, and 36% not knowing/answering for NHP‐to‐human transmission; 34%, 21%, and 45% respectively for human‐to‐NHP transmission), with no significant differences between YF‐positive and YF‐negative areas (χ^2^ = 1.44, df = 2, *p* > 0.05 for NHP‐to‐human transmission; χ^2^ = 0.46, df = 2, *p* > 0.05 for human‐to‐NHP transmission; Figure 5).

No evidence of problematic multicollinearity was detected among predictors across any model (all adjusted GVIFs < 2.1; see Supporting Information S1 Table S12).

## 4 Discussion

Negative experiences, such as zoonotic disease outbreaks involving wildlife, can lower human tolerance by increasing perceived costs of coexistence. However, respondents living in municipalities with previous YF outbreaks did not report consistently more negative perceptions of either species at the time of the study. Instead, perceptions of howler monkeys were more favorable among men, respondents reporting more frequent sightings, and respondents with higher education levels. Furthermore, fear of disease transmission by NHPs, including YF, was similar across YF‐positive and YF‐negative municipalities. Together, these findings suggest that socio‐demographic and experiential factors may contribute importantly to shaping perceptions of NHPs. Nevertheless, since the interviews were conducted after major outbreak events ended, our results cannot exclude the possibility that negative perceptions toward NHPs increase temporarily during active YF outbreaks and diminish over time.

Since more frequent encounters with a stimulus generally foster more positive attitudes (Zajonc 1968), including toward wildlife such as bats (Boso et al. 2021), human perceptions of NHPs are likely influenced by how often people encounter them. Supporting this hypothesis, we found overall positive perceptions of marmosets, which were both more frequently reported and more accurately identified than howler monkeys. In the study region, high encounter rates with marmosets may reflect a combination of ecological and socio‐environmental factors, including their frequent use of human‐modified environments (Teixeira et al. 2015), ecological plasticity (Grande et al. 2020), and anthropogenic changes such as urban expansion and habitat disturbance that tend to favor generalist species (Thatcher et al. 2021), and the widespread establishment of invasive or hybrid *Callithrix* populations in the Atlantic Forest, which may further increase their local prevalence (Vale et al. 2020). Greater familiarity may therefore help explain the consistently positive perceptions of marmosets, while more frequent sightings of howler monkeys were also associated with more positive perceptions, suggesting an association between frequent encounters and more positive perceptions or tolerance. However, these patterns should be interpreted within the regional socio‐ecological context of the study. Although howler monkeys use urban environments in southern Brazil (Corrêa et al. 2018), Paraguay (Alesci et al. 2022) or northern Argentina (Citon et al. 2025), in the study areas they appear to act as forest‐dependent species (Grande et al. 2020), likely limiting everyday human encounters. Consistent with this interpretation, higher proportions of “no opinion” responses were reported for howler monkey perception questions compared to marmosets, reinforcing the role of limited exposure in shaping weaker or less formed attitudes.

The lack of significant predictors for marmoset perceptions further suggests that their broad distribution and abundance in human‐modified landscapes generate a relatively uniform positive public view, masking subtler perceptual differences, even though they are recognized as rabies reservoirs (Kotait et al. 2018). Nevertheless, repeated encounters alone cannot guarantee positive perceptions, as interactions involving negative experiences may instead amplify perceived costs and reinforce negative attitudes (Amman et al. 2014; Kansky et al. 2014; Kansky et al. 2016; Franchini et al. 2021). The positive perceptions associated with high encounter frequencies may reflect not only exposure itself, but also species‐specific traits of marmosets, such as small size and non‐threatening appearance, which may contribute to more positive attitudes towards the species.

In Brazil, fear of YF transmission might be expected to amplify risk perceptions and foster reactive negative behaviors towards NHPs (Bicca‐Marques and de Freitas 2010). However, our results revealed predominantly positive perceptions of marmosets and howler monkeys, even in municipalities with previous YF outbreaks, despite most respondents expressing fear of zoonotic transmission. Likewise, neither the perceptions of NHPs nor fear of disease transmission exhibited consistent differences even when distinguishing municipalities with human‐YF cases from those with NHP‐associated YF occurrence. It is also possible that beliefs regarding NHPs as YF transmitters become amplified during active outbreaks and diminish over time, such that previous local experience with YF does not necessarily result in long‐term changes in perception. Informal comments collected during interviews additionally suggested that speculations, misinformation, and locally transmitted beliefs may play an important role in shaping negative reactions towards NHPs during outbreak periods, including reports of people poisoning or attacking monkeys due to fears of disease transmission. The generally positive perceptions observed here should be interpreted cautiously, as potential biases such as the overrepresentation of more interested respondents (non‐participation bias), the tendency to give socially acceptable answers (social desirability bias), or the recruitment of urban respondents near forest fragments where human‐NHP encounters are more frequent, may have contributed to over‐reporting of favorable attitudes (Nuno and St. John 2014). Overall, our results suggest that relatively stable perceptions measured outside outbreak periods may be insufficient to explain acute negative behavioral responses, such as NHP killings, during YF epidemics. Therefore, future research should focus on longitudinal or real‐time studies conducted during outbreak periods.

Our analyses suggested that higher education levels were associated with more positive perceptions of howler monkeys. However, this association should be interpreted cautiously, as education level was unevenly distributed among municipalities, meaning that the observed association may partly reflect between‐municipality differences rather than individual educational level. The interaction analysis provided no evidence that YF status moderated the education association. Nevertheless, these results remain consistent with previous research showing that education reduces risk perception and fosters more favorable attitudes toward wildlife often regarded as threatening, such as bats, foxes, and wolves (Kimmig et al. 2020; Boso et al. 2021; Koshkina et al. 2026). Education may enhance understanding of the ecological roles of NHPs and improve knowledge of zoonotic transmission pathways, potentially reducing misconceptions and the perceived costs of coexistence, thereby reinforcing perceived benefits and tolerance (Puri et al. 2024; Koshkina et al. 2026). In addition, respondents with higher education levels may perceive greater control over disease prevention or have higher trust in public health agencies, which could reduce perceived risk. Previous studies have also shown that tolerance towards wildlife is shaped not only by knowledge, but also by emotional and contextual factors (Kansky et al. 2016). Likewise, municipalities experiencing human‐YF outbreaks may also differ in factors such as media exposure, public health communication, or urban and academic contexts, which could additionally shape perceptions of disease risk and coexistence. The observed association between education and perception therefore remains broadly consistent with previous studies linking education and knowledge with lower perceived risk and greater tolerance toward wildlife‐associated hazards (Hanisch‐Kirkbride et al. 2013; Bruskotter and Wilson 2014). Altogether, these patterns suggest that communication and coexistence strategies may need to be adapted to different socio‐demographic groups and outbreak contexts. Beyond improving knowledge about disease transmission, increasing public trust in health agencies and reinforcing the perception that disease risk can be effectively managed may also help reduce reactive negative responses towards NHPs during outbreaks.

In our study, women exhibited less favorable perceptions of howler monkeys and higher perceived risk of disease transmission (i.e., greater fear) compared to men. This pattern has been consistently documented across diverse taxa and contexts (Hanisch‐Kirkbride et al. 2013; Kimmig et al. 2020; Boso et al. 2021; Koshkina et al. 2025). Gender‐biased perceptions of wildlife risk often arise from both differential exposure and innate perceptual differences shaped by social and cultural factors (Gore and Kahler 2012). In some contexts, gender‐specific roles may influence opportunities for contact with wildlife. For example, men in Brazil are more often engaged in outdoor or forest‐related activities, potentially increasing familiarity with local fauna and encounters with forest‐dependent species such as howler monkeys (Bitzer et al. 2024). However, these mechanisms are likely context‐dependent and may vary across urban and rural settings. Women's lower familiarity with wildlife in some socio‐ecological contexts, combined with heightened risk perception, may also contribute to increased fear and lower confidence in managing potential risks (Zinn et al. 2008; Puri et al. 2024), particularly for a species with a large size and the loudest howl among terrestrial animals. Interview comments also suggested that local knowledge and beliefs surrounding howler monkey vocalizations and behavior (e.g., associating vocal activity with incoming rain periods or considering possible feces‐throwing interpreted as aggressive behavior) may influence how these species are perceived in some communities. Alternatively, men may underreport fear in questionnaires due to social norms discouraging emotional expression. Recognizing these gender‐based differences may help improve the effectiveness of educational and outreach strategies across local contexts.

Despite providing important insights on human perceptions of NHPs, our study has several limitations that should guide future research. Because perceptions are strongly context‐dependent and interviews were conducted after major YF outbreaks ended, our results cannot be generalized beyond the studied area or used to assess potential short‐term changes in perceptions or reactive behaviors occurring during active outbreaks. For example, patterns may differ in southern Brazil outside the range of *Callithrix* species, such as Rio Grande do Sul, where howler monkeys were more heavily persecuted during YF outbreaks, as they were mistakenly perceived as disease transmitters due to their high susceptibility to YF. In addition, YF‐status varied at the municipality level across only seven municipalities, with three classified as YF‐positive, resulting in limited effective degrees of freedom for the YF contrast (7.33 for marmosets, 9.01 for howler monkeys, see Supporting Information S1 Table S5). This cluster‐level structure limits the precision and statistical power of inference regarding the association between YF occurrence and NHP perceptions, independently of the socio‐demographic imbalance. The distribution of some socio‐demographic variables also differed between YF‐positive and YF‐negative municipalities, particularly education level and gender. Although these variables were included as fixed effects and additional sensitivity analyses yielded qualitatively similar results, this structural imbalance may still limit the extent to which individual‐level effects can be disentangled from differences between municipalities. Moreover, municipalities classified as human‐YF and NHP‐YF also differed in broader contextual characteristics, including urbanization and respondent composition, which could not be fully disentangled from YF exposure in our analyses. Consequently, although we found no consistent evidence that YF occurrence was associated with perceptions of NHPs or disease risk, these null results should be interpreted cautiously given the limited number of municipality‐level clusters and the remaining contextual differences between YF‐positive and YF‐negative municipalities. Other potentially relevant predictors of perception, including occupation, income or religion, were not included in our survey. Additionally, our approach provided limited insight into the broader social and cultural processes underlying human perceptions of NHPs. Non‐participation and social desirability biases may also have led to an underestimation of negative attitudes; although difficult to overcome, these could be mitigated by triangulating interviews with systematic field observations or participatory monitoring.

## 5 Conclusions

Our findings have important implications for management decisions regarding human‐NHP coexistence, showing that generally positive attitudes towards marmosets and howler monkeys persist across municipalities with and without historic YF outbreaks. However, coexistence, conservation and public health communication campaigns should account for socio‐demographic and contextual differences influencing wildlife risk perception, particularly during zoonotic outbreaks. Future research should consider additional factors, including social trust in governmental agencies, misinformation dynamics, and temporal changes in perceptions during outbreak periods, to better understand the drivers of NHP killings during YF outbreaks and the persistence of negative attitudes over time.

## Author Contributions

**Cristina Caparrós‐Vallcorba:** conceptualization, methodology, data curation, investigation, formal analysis, funding acquisition, project administration, resources, writing – original draft, writing – review and editing. **Filipe Vieira Santos de Abreu:** conceptualization, methodology, investigation, funding acquisition, writing – review and editing, project administration. **Danilo Simonini‐Teixeira:** methodology, supervision, funding acquisition, writing – review and editing. **Waldney Pereira‐Martins:** funding acquisition, writing – review and editing. **Antonio Victor Veloso‐Ramos:** methodology, writing – review and editing. **Sofía Bernal‐Valle:** methodology, writing – review and editing. **Mariana Martins‐Carvalho:** methodology. **Sandy Micaele Aquino‐Teixeira:** methodology. **Naiane Jardim:** methodology. **Thayná Senario:** methodology. **Hermes P. Almeida:** methodology, resources. **Claudio Azat:** methodology, supervision, writing – review and editing. **Julio A Benavides:** conceptualization, data curation, formal analysis, supervision, funding acquisition, writing – review and editing, writing – original draft.

## Conflicts of Interest

The authors declare no conflicts of interest.

## Supporting information


Supporting File 1

Supporting File 2

## Data Availability

The datasets generated and analyzed during the current study contain sensitive information about human participants and cannot be shared publicly due to ethical and privacy restrictions. Aggregated data supporting the main findings are available within the article and its supplementary materials.
