# Supplementary material for: Yellow Fever Outbreaks Are Not Associated With Long‐Term Negative Human Perceptions of Wild Primates in Brazil
Source: Am J Primatol. 2026 Oct 21;88(10):e70219. doi: 10.1002/ajp.70219 (PMC13685054; doi:10.1002/ajp.70219)
Supplement: Supplementary file 1 [file AJP-88-e70219-s001.docx]

**Supporting Information S1:**

**Yellow fever outbreaks are not associated with long-term negative human perceptions of wild primates in Brazil**

## **Table S1**. Differentiation of urban and rural areas based on administrative, morphological and functional criteria. Information extracted from the Instituto Brasileiro de Geografia e Estatística – IBGE (IBGE, 2023).

| **Urban areas** | **Rural areas** |
| --- | --- |
| - Urban areas with high density of buildings. - Rural agglomerations without private or corporate character not linked to a single landowner and characterized by the existence of commerce and services. - Isolated urban areas and rural agglomerations of urban expansion that are detached from the urban area. Includes housing complexes and condominiums even in rural areas. | - Urban areas with low building density, with urban expansion processes, uninhabited green areas, among others. - Rural agglomerations linked to a single landowner (e.g., agricultural companies, agribusiness, etc.). - Hamlet as rural agglomeration without urban services or facilities that define villages, not linked to a single owner. - Areas of rural use excluding agglomerations, characterized by the dispersion of dwellings and the usual presence of agricultural establishments. |

Ministério da Saúde do Brasil. (2023). *Febre Amarela em humanos e primatas não-humanos - 1994 a 2023*. Sistema Único Da Saúde.

## **Table S2**. List of municipalities screened during site selection with occurrence of Yellow Fever in non-human primates, humans or both, and the year of the outbreak in brackets (Ministério da Saúde do Brasil, 2023).

## **YF-positive municipalities**

## Varginha (2018)

## Juramento (2020)

## Unaí (2020)

## São João da Lagoa (2021)

## Icaraí de Minas (2021)

## Belo Horizonte (2021)

## Coração de Jesus (2021)

## Ubaí (2021, 2022)

## Curvelo (2021)

## Brasília de Minas (2021, 2022)

Itapagipe (2022)

Uberaba (2022)

IBGE. (2023). *Malha de setores censitários / IBGE*. https://biblioteca.ibge.gov.br/index.php/biblioteca-catalogo?view=detalhes&id=2102138

## **Table S3**. Summary of respondent demographics.

Abbreviations: BM for Brasília de Minas; BH for Belo Horizonte. “No secondary education” includes respondents with no education, incomplete or complete fundamental education, and incomplete secondary education. “Secondary education” includes respondents with complete secondary education. “More than secondary education” includes respondents with incomplete and complete university degrees and post-graduation studies.

|  | **Minas Gerais** | | | | | **Bahía** | | **Total** |
| --- | --- | --- | --- | --- | --- | --- | --- | --- |
|  | **Ubaí** | **BM** | **BH** | **Almenara** | **Salinas** | **Macaraní** | **Itapetinga** |  |
| **Men** | 32 | 15 | 24 | 41 | 41 | 45 | 45 | **243** |
| **Women** | 53 | 21 | 30 | 35 | 32 | 30 | 30 | **231** |
| **Mean age (years old)** | 48 | 46 | 40 | 52 | 50 | 48 | 47 | **49** |
| **Urban** | 41 | 36 | 54 | 36 | 35 | 39 | 36 | **277** |
| **Rural** | 44 | 0 | 0 | 40 | 38 | 36 | 39 | **197** |
| **No secondary education** | 54 | 12 | 7 | 52 | 58 | 55 | 52 | **290** |
| *No education* | *10* | *0* | *0* | *10* | *5* | *11* | *11* | *47* |
| *Incomplete fundamental education* | *30* | *8* | *4* | *30* | *43* | *26* | *22* | *163* |
| *Complete fundamental education* | *11* | *3* | *1* | *4* | *6* | *8* | *8* | *41* |
| *Incomplete secondary education* | *3* | *1* | *2* | *8* | *4* | *10* | *11* | *39* |
| **Secondary education** | 18 | 11 | 12 | 14 | 10 | 15 | 16 | **96** |
| **More than secondary education** | 13 | 13 | 35 | 10 | 5 | 5 | 7 | **88** |
| *Incomplete superior education* | *2* | *4* | *23* | *4* | *1* | *1* | *0* | *35* |
| *Complete superior education* | *10* | *4* | *9* | *5* | *4* | *4* | *6* | *42* |
| *Post graduation studies* | *1* | *5* | *3* | *1* | *0* | *0* | *1* | *11* |
| **Total** | 85 | 36 | 54 | 76 | 73 | 75 | 75 | **474** |
|  | YF-positive | | | YF-negative | | | |  |

| **Table S4**. Items constituting the elements used to calculate the human perception index, and their related responses. All items included a “No opinion” answer. | | | | | |
| --- | --- | --- | --- | --- | --- |
| **Perception items** | **1** | **2** | **3** | **4** | **5** |
| If I could choose the quantity of marmosets / howler monkeys in the region will… | Diminish a lot | Diminish | Be maintained | Increase | Increase a lot |
| If marmosets / howler monkeys were to disappear from the region, I would feel… | Very happy | Happy | Indifferent | Sad | Very sad |
| When I see marmosets / howler monkeys, I… | Hate them | Don’t like them | I don’t care | Like them | Love them |
| Marmosets / Howler monkeys are… | A big problem | A problem | I am indifferent to them | A benefit | A big benefit |
| My experiences with marmosets / howler monkeys are… | Always negative | Generally negative | Indifferent | Generally positive | Always positive |
| I believe that marmosets / howler monkeys have a ________ effect on wildlife and nature. | Very harmful | Harmful | No effect | Beneficial | Very beneficial |

|  |  |
| --- | --- |
|  |  |
|  |  |
| **Figure S1.** Residual diagnostic plots for the final linear mixed-effect models evaluating human perception of marmosets and howler monkeys. | |

| **Table S5.** Output from full additive model LMMs on squared perception indices for marmosets and howler monkeys. Sighting frequencies: high frequency for daily or weekly sightings; medium frequency for monthly or occasional sightings, from once to four times per year; and low frequency for less than once per year, including those who had seen them only once, never, or did not know/declined to answer. Abbreviations: SE for Standard Error. | | | | | | |
| --- | --- | --- | --- | --- | --- | --- |
|  | | **Fixed effects** | **Estimate** | **SE** | **df** | ***p*** |
| **Marmosets** | **n = 474; AIC = 2840.0** | | | | | |
|  | Intercept |  | 15.52 | 1.43 | 39.55 | **<0.001***** |
|  | Urbanization | (urban) | 0.01 | 1.49 | 470.75 | 0.989 |
|  | YF-occurrence | (YF-positive) | -0.72 | 1.42 | 7.33 | 0.628 |
|  | Age (years old) | (27-39) | -0.44 | 0.81 | 461.22 | 0.593 |
|  |  | (40-52) | 0.17 | 0.77 | 465.17 | 0.825 |
|  |  | (53-64) | -0.42 | 0.83 | 464.27 | 0.616 |
|  |  | (> 64) | -0.35 | 0.84 | 462.17 | 0.675 |
|  | Education | (secondary education) | 0.64 | 0.58 | 458.93 | 0.267 |
|  |  | (more than secondary education) | 1.27 | 0.67 | 467.55 | **0.061 .** |
|  | Gender | (female) | -0.43 | 0.44 | 463.88 | 0.321 |
|  | Sight frequency | (high frequency) | 0.16 | 0.77 | 462.08 | 0.838 |
|  |  | (medium frequency) | -0.94 | 0.80 | 464.25 | 0.243 |

_________________________________________________________________________

| **Howler monkeys** | **n = 432; AIC = 2604.5** | | | | | |
| --- | --- | --- | --- | --- | --- | --- |
|  | Intercept |  | 13.11 | 1.09 | 45.91 | **<0.001***** |
|  | Urbanization | (urban) | 0.27 | 0.52 | 429.52 | 0.606 |
|  | YF-occurrence | (YF-positive) | -0.70 | 0.89 | 9.01 | 0.456 |
|  | Age (years old) | (27-39) | 0.34 | 0.87 | 423.35 | 0.692 |
|  |  | (40-52) | 1.51 | 0.80 | 425.48 | **0.061 .** |
|  |  | (53-64) | 0.05 | 0.88 | 424.77 | 0.958 |
|  |  | (> 64) | 0.84 | 0.87 | 424.88 | 0.335 |
|  | Education | (secondary education) | 0.75 | 0.62 | 419.80 | 0.228 |
|  |  | (more than secondary education) | 2.10 | 0.71 | 417.81 | **0.003 **** |
|  | Gender | (female) | -1.52 | 0.48 | 425.29 | **0.002 **** |
|  | Sight frequency | (high frequency) | 2.46 | 1.02 | 417.96 | **0.016 *** |
|  |  | (medium frequency) | 1.10 | 0.68 | 410.45 | 0.109 |

**Sensitivity analyses: Table S6, S7, S8 and S9**

**Table S6.** Sensitivity analyses of the howler monkey perception model with details of the full additive model, the education-restricted model (exclusion of respondents with more-than-secondary education), and the interaction model (YF-status x education).

| **Fixed effect** | | **Estimate** | **SE** | ***p*** |
| --- | --- | --- | --- | --- |
| **Full additive model** | **n = 432; AIC = 2604.5** | | | |
|  | Intercept | 13.11 | 1.09 | **<0.001***** |
|  | Urbanization (ref: rural) | 0.27 | 0.52 | 0.606 |
|  | YF status (ref: YF-negative) | −0.70 | 0.89 | 0.456 |
|  | Age 27–39 (ref. 18–26) | 0.34 | 0.87 | 0.692 |
|  | Age 40–52 (ref. 18–26) | 1.51 | 0.80 | 0.061 |
|  | Age 53–64 (ref. 18–26) | 0.05 | 0.88 | 0.958 |
|  | Age >64 (ref. 18–26) | 0.84 | 0.87 | 0.335 |
|  | Education: secondary (ref. less than secondary) | 0.75 | 0.62 | 0.228 |
|  | Education: more than secondary (ref. less than secondary) | 2.10 | 0.71 | **0.003**** |
|  | Gender: female (ref. male) | −1.52 | 0.48 | **0.002**** |
|  | Sighting frequency: medium (ref. low) | 1.10 | 0.68 | 0.109 |
|  | Sighting frequency: high (ref. low) | 2.46 | 1.02 | **0.016*** |
| **Education-restricted model** | **n = 351; AIC = 2152.4** | | | |
|  | Intercept | 13.47 | 1.26 | **<0.001***** |
|  | Urbanization (ref: rural) | 0.34 | 0.58 | 0.557 |
|  | YF status (ref: YF-negative) | −1.08 | 1.00 | 0.307 |
|  | Age 27–39 (ref. 18–26) | 0.37 | 1.12 | 0.742 |
|  | Age 40–52 (ref. 18–26) | 1.62 | 1.05 | 0.126 |
|  | Age 53–64 (ref. 18–26) | −0.12 | 1.11 | 0.917 |
|  | Age >64 (ref. 18–26) | 0.58 | 1.11 | 0.600 |
|  | Education: secondary (ref. less than secondary) | 0.74 | 0.67 | 0.273 |
|  | Gender: female (ref. male) | −1.67 | 0.56 | **0.003**** |
|  | Sighting frequency: medium (ref. low) | 0.93 | 0.76 | 0.222 |
|  | Sighting frequency: high (ref. low) | 2.48 | 1.21 | **0.042*** |
| **Interaction model** | **n = 432; AIC = 2607.9** | | | |
|  | Intercept | 13.11 | 1.09 | **<0.001***** |
|  | Urbanization (ref: rural) | 0.28 | 0.52 | 0.591 |
|  | YF status (ref: YF-negative) | -1.03 | 1.02 | 0.328 |
|  | Age 27–39 (ref. 18–26) | 0.40 | 0.87 | 0.642 |
|  | Age 40–52 (ref. 18–26) | 1.57 | 0.81 | 0.054 |
|  | Age 53–64 (ref. 18–26) | 0.13 | 0.89 | 0.885 |
|  | Age >64 (ref. 18–26) | 0.92 | 0.88 | 0.299 |
|  | Gender: female (ref. male) | −1.50 | 0.48 | **0.002**** |
|  | Sighting frequency: medium (ref. low) | 1.09 | 0.68 | 0.112 |
|  | Sighting frequency: high (ref. low) | 2.45 | 1.02 | **0.017*** |
|  | Education: secondary (ref. less than secondary) | 0.65 | 0.78 | 0.405 |
|  | Education: more than secondary (ref. less than secondary) | 1.56 | 1.05 | 0.139 |
|  | Secondary education × YF status (ref: YF-negative) | 0.42 | 1.27 | 0.743 |
|  | More than secondary education × YF status (ref: YF-negative) | 1.03 | 1.41 | 0.468 |

**Note: The full additive model included all predefined predictors (urbanization, YF status, gender, education level, age group, and howler monkey sighting frequency). The restricted model used the same specification after excluding respondents with more-than-secondary education. The interaction model used the full additive specification with an education × YF-status interaction. AIC values of the full additive and interaction models were calculated using the same dataset (n = 432); the restricted model was fitted to a different dataset (n = 351) and its AIC was therefore not directly compared with the other models. Significance codes: * p < 0.05; ** p < 0.01; *** p < 0.001.*

**Table S7.** Sensitivity analyses alternative classification of yellow fever (YF) occurrences. Results are shown for marmoset and howler monkey perception models, and for models of beliefs and fear regarding disease transmission from NHPs.

| **Fixed effect** | | **Estimate** | **SE** | ***p*** |
| --- | --- | --- | --- | --- |
| **Marmoset perception –**  **full LMM** | **n = 474; AIC = 2839.40** | | | |
|  | Intercept | 15.48 | 1.34 | **<0.001***** |
|  | Urbanization (ref: rural) | −0.01 | 0.49 | 0.979 |
|  | YF status: NHP-YF (ref. no-YF) | −1.81 | 1.35 | 0.217 |
|  | YF status: human-YF (ref. no-YF) | 1.55 | 1.72 | 0.395 |
|  | Age 27–39 (ref. 18–26) | −0.38 | 0.81 | 0.641 |
|  | Age 40–52 (ref. 18–26) | 0.24 | 0.77 | 0.751 |
|  | Age 53–64 (ref. 18–26) | −0.35 | 0.83 | 0.679 |
|  | Age >64 (ref. 18–26) | −0.30 | 0.84 | 0.718 |
|  | Education: secondary (ref. less than secondary) | 0.64 | 0.58 | 0.269 |
|  | Education: more than secondary (ref. less than secondary) | 1.19 | 0.67 | 0.079 |
|  | Gender: female (ref. male) | −0.42 | 0.44 | 0.333 |
|  | Sighting frequency: medium (ref. low) | −0.94 | 0.80 | 0.238 |
|  | Sighting frequency: high (ref. low) | 0.14 | 0.77 | 0.856 |
| **Howler monkey perception –**  **full LMM** | **n = 432; AIC = 2605.10** | | | |
|  | Intercept | 13.06 | 1.06 | **<0.001***** |
|  | Urbanization (ref: rural) | 0.27 | 0.52 | 0.608 |
|  | YF status: NHP-YF (ref. no-YF) | −1.32 | 0.96 | 0.200 |
|  | YF status: human-YF (ref. no-YF) | 0.34 | 1.22 | 0.784 |
|  | Age 27–39 (ref. 18–26) | 0.41 | 0.87 | 0.634 |
|  | Age 40–52 (ref. 18–26) | 1.59 | 0.81 | 0.050 |
|  | Age 53–64 (ref. 18–26) | 0.12 | 0.88 | 0.888 |
|  | Age >64 (ref. 18–26) | 0.91 | 0.88 | 0.301 |
|  | Education: secondary (ref. less than secondary) | 0.75 | 0.62 | 0.231 |
|  | Education: more than secondary (ref. less than secondary) | 1.97 | 0.72 | **0.007**** |
|  | Gender: female (ref. male) | −1.46 | 0.48 | **0.002**** |
|  | Sighting frequency: medium (ref. low) | 1.22 | 0.69 | 0.078 |
|  | Sighting frequency: high (ref. low) | 2.66 | 1.03 | **0.010*** |
| **Belief of disease transmission – multinomial GLMM**  reference outcome = Yes (s) | **n = 474; AIC = 757.29**  *No vs Yes* | | | |
|  | Intercept | −1.85 | 0.85 | **0.030*** |
|  | Urbanization (ref: rural) | 0.48 | 0.39 | 0.218 |
|  | YF status: NHP-YF (ref. no-YF) | 0.17 | 0.46 | 0.707 |
|  | YF status: human-YF (ref. no-YF) | 0.59 | 0.54 | 0.274 |
|  | Age 27–39 (ref. 18–26) | 0.24 | 0.62 | 0.698 |
|  | Age 40–52 (ref. 18–26) | 0.42 | 0.58 | 0.475 |
|  | Age 53–64 (ref. 18–26) | −0.13 | 0.67 | 0.842 |
|  | Age >64 (ref. 18–26) | 0.21 | 0.63 | 0.735 |
|  | Education: secondary (ref. less than secondary) | −0.31 | 0.46 | 0.499 |
|  | Education: more than secondary (ref. less than secondary) | −0.38 | 0.49 | 0.438 |
|  | Gender: female (ref. male) | −0.36 | 0.33 | 0.284 |
|  | Marmoset sighting frequency: medium (ref. high) | −0.29 | 0.42 | 0.486 |
|  | Marmoset sighting frequency: low (ref. high) | 0.40 | 0.50 | 0.422 |
|  | Howler sighting frequency: medium (ref. high) | −0.93 | 0.73 | 0.207 |
|  | Howler sighting frequency: low (ref. high) | −0.42 | 0.66 | 0.520 |
|  | **n = 474; AIC = 757.29**  *Don't know/No answer vs Yes* | | | |
|  | Intercept | −2.02 | 0.90 | **0.025*** |
|  | Urbanization (ref: rural) | −0.52 | 0.29 | 0.074 |
|  | YF status: NHP-YF (ref. no-YF) | 0.17 | 0.37 | 0.652 |
|  | YF status: human-YF (ref. no-YF) | 0.34 | 0.53 | 0.518 |
|  | Age 27–39 (ref. 18–26) | −0.20 | 0.48 | 0.684 |
|  | Age 40–52 (ref. 18–26) | −0.30 | 0.46 | 0.514 |
|  | Age 53–64 (ref. 18–26) | −0.43 | 0.50 | 0.388 |
|  | Age >64 (ref. 18–26) | 0.13 | 0.48 | 0.780 |
|  | Education: secondary (ref. less than secondary) | 0.37 | 0.32 | 0.251 |
|  | Education: more than secondary (ref. less than secondary) | −1.06 | 0.53 | **0.046*** |
|  | Gender: female (ref. male) | −0.22 | 0.27 | 0.405 |
|  | Marmoset sighting frequency: medium (ref. high) | 0.19 | 0.31 | 0.543 |
|  | Marmoset sighting frequency: low (ref. high) | 0.62 | 0.41 | 0.126 |
|  | Howler sighting frequency: medium (ref. high) | 0.67 | 0.82 | 0.416 |
|  | Howler sighting frequency: low (ref. high) | 1.05 | 0.80 | 0.186 |
| **Fear of disease transmission — multinomial GLMM** | **n = 474; AIC = 756.13**  *No vs Yes* | | | |
|  | Intercept | 0.03 | 0.36 | 0.935 |
|  | Urbanization (ref: rural) | 0.00 | 0.22 | 0.988 |
|  | YF status: NHP-YF (ref. no-YF) | −0.30 | 0.24 | 0.221 |
|  | YF status: human-YF (ref. no-YF) | 0.69 | 0.37 | 0.059 |
|  | Age 27–39 (ref. 18–26) | −0.29 | 0.37 | 0.427 |
|  | Age 40–52 (ref. 18–26) | −0.46 | 0.35 | 0.191 |
|  | Age 53–64 (ref. 18–26) | −0.50 | 0.39 | 0.195 |
|  | Age >64 (ref. 18–26) | −0.10 | 0.38 | 0.793 |
|  | Education: secondary (ref. less than secondary) | 0.29 | 0.27 | 0.269 |
|  | Education: more than secondary (ref. less than secondary) | 0.40 | 0.31 | 0.201 |
|  | Gender: female (ref. male) | −0.64 | 0.20 | **0.001**** |
|  | **n = 474; AIC = 756.13**  *Don't know/No answer vs Yes* | | | |
|  | Intercept | −1.80 | 0.86 | **0.037*** |
|  | Urbanization (ref: rural) | −1.18 | 0.72 | 0.101 |
|  | YF status: NHP-YF (ref. no-YF) | −0.60 | 0.82 | 0.462 |
|  | YF status: human-YF (ref. no-YF) | 0.87 | 1.11 | 0.433 |
|  | Age 27–39 (ref. 18–26) | −1.02 | 0.99 | 0.299 |
|  | Age 40–52 (ref. 18–26) | −1.54 | 0.99 | 0.120 |
|  | Age 53–64 (ref. 18–26) | −0.49 | 0.90 | 0.588 |
|  | Age >64 (ref. 18–26) | −0.91 | 1.02 | 0.372 |
|  | Education: secondary (ref. less than secondary) | −0.04 | 0.86 | 0.964 |
|  | Education: more than secondary (ref. less than secondary) | 0.56 | 0.90 | 0.534 |
|  | Gender: female (ref. male) | 0.07 | 0.59 | 0.909 |

**Note: YF occurrence was classified into three categories: no-YF, NHP-YF, and human-YF, with no-YF as the reference category. The full perception models were re-fitted using this three-level YF-status variable. For multinomial models, coefficients are presented for “No” and “Don’t know / No answer” relative to “Yes”. Estimates represent log-odds coefficients. Reference categories are indicated in the table. Significance codes: * p < 0.05; ** p < 0.01; *** p < 0.001.*

**Table S8.** Sensitivity analyses of perception models restricted to respondents who correctly identified at least one focal species image.

|  | | **Fixed effect** | **Estimate** | **SE** | ***p*** |
| --- | --- | --- | --- | --- | --- |
| **Marmoset perception**  **— full LMM** | **n = 449; AIC = 2686.90.** | | | | |
|  | Intercept | | 15.39 | 1.46 | **<0.001***** |
|  | Urbanization (ref: rural) | | −0.00 | 0.50 | 1.000 |
|  | YF status (ref: YF-negative) | | −0.56 | 1.33 | 0.687 |
|  | Age 27–39 (ref. 18–26) | | 0.08 | 0.85 | 0.928 |
|  | Age 40–52 (ref. 18–26) | | 0.67 | 0.80 | 0.405 |
|  | Age 53–64 (ref. 18–26) | | −0.02 | 0.86 | 0.984 |
|  | Age >64 (ref. 18–26) | | 0.08 | 0.87 | 0.931 |
|  | Education: secondary (ref. less than secondary) | | 0.63 | 0.59 | 0.286 |
|  | Education: more than secondary (ref. less than secondary) | | 1.17 | 0.69 | 0.090 |
|  | Gender: female (ref. male) | | −0.31 | 0.44 | 0.484 |
|  | Sighting frequency: medium (ref. low) | | −1.23 | 0.86 | 0.154 |
|  | Sighting frequency: high (ref. low) | | −0.23 | 0.83 | 0.785 |
| **Howler monkey perception — full LMM** | **n = 163; AIC = 998.10** | | | | |
|  | Intercept | | 13.10 | 1.55 | **<0.001***** |
|  | Urbanization (ref: rural) | | 0.36 | 0.85 | 0.675 |
|  | YF status (ref: YF-negative) | | −0.81 | 1.03 | 0.434 |
|  | Age 27–39 (ref. 18–26) | | 0.90 | 1.48 | 0.543 |
|  | Age 40–52 (ref. 18–26) | | 2.32 | 1.31 | 0.078 |
|  | Age 53–64 (ref. 18–26) | | 1.14 | 1.41 | 0.422 |
|  | Age >64 (ref. 18–26) | | 2.47 | 1.39 | 0.078 |
|  | Education: secondary (ref. less than secondary) | | 1.07 | 1.06 | 0.312 |
|  | Education: more than secondary (ref. less than secondary) | | 2.85 | 1.11 | **0.011*** |
|  | Gender: female (ref. male) | | −2.44 | 0.81 | **0.003**** |
|  | Sighting frequency: medium (ref. low) | | −0.00 | 0.93 | 0.998 |
|  | Sighting frequency: high (ref. low) | | 0.48 | 1.26 | 0.700 |

**Note: Sensitivity analyses were restricted to respondents who correctly identified at least one focal species image, regardless of whether additional incorrect images were also selected. The full model specification was retained for these sensitivity analyses. YF status was not significantly associated with perceptions of either species. Significance codes: * p < 0.05; ** p < 0.01; *** p < 0.001.*

**Table S9.** Sensitivity analyses of howler monkey perception models fitted separately for urban and rural respondents.

|  | | **Fixed effect** | **Estimate** | **SE** | ***p*** |
| --- | --- | --- | --- | --- | --- |
| **Rural full LMM** | **n = 191; AIC = 1158.10** | | | | |
|  | Intercept | | 13.23 | 1.57 | **<0.001***** |
|  | YF status (ref: YF-negative) | | −1.89 | 1.05 | 0.074 |
|  | Age 27–39 (ref. 18–26) | | 0.01 | 1.51 | 0.993 |
|  | Age 40–52 (ref. 18–26) | | 1.32 | 1.44 | 0.360 |
|  | Age 53–64 (ref. 18–26) | | 0.36 | 1.48 | 0.808 |
|  | Age >64 (ref. 18–26) | | 1.17 | 1.56 | 0.453 |
|  | Education: secondary (ref. less than secondary) | | 0.90 | 0.97 | 0.355 |
|  | Education: more than secondary (ref. less than secondary) | | 2.09 | 1.40 | 0.137 |
|  | Gender: female (ref. male) | | −1.57 | 0.72 | **0.031*** |
|  | Sighting frequency: medium (ref. low) | | 1.02 | 0.91 | 0.268 |
|  | Sighting frequency: high (ref. low) | | 3.56 | 1.34 | **0.009**** |
| **Urban full LMM** | **n = 241; AIC = 1474.90** | | | | |
|  | Intercept | | 13.57 | 1.28 | **<0.001***** |
|  | YF status (ref: YF-negative) | | −0.73 | 1.14 | 0.539 |
|  | Age 27–39 (ref. 18–26) | | 0.49 | 1.09 | 0.653 |
|  | Age 40–52 (ref. 18–26) | | 1.59 | 0.99 | 0.110 |
|  | Age 53–64 (ref. 18–26) | | −0.30 | 1.15 | 0.797 |
|  | Age >64 (ref. 18–26) | | 0.38 | 1.09 | 0.727 |
|  | Education: secondary (ref. less than secondary) | | 0.61 | 0.84 | 0.472 |
|  | Education: more than secondary (ref. less than secondary) | | 1.82 | 0.90 | **0.044*** |
|  | Gender: female (ref. male) | | −1.34 | 0.65 | **0.040*** |
|  | Sighting frequency: medium (ref. low) | | 1.68 | 1.71 | 0.327 |
|  | Sighting frequency: high (ref. low) | | 1.34 | 1.08 | 0.216 |

*Both models included municipality and researcher as random effects; the municipality random effect had zero variance in the rural model. The YF-status estimate in the rural model should not be interpreted because only one rural municipality (Ubaí) was YF-positive.*

*Significance codes: * p < 0.05; ** p < 0.01; *** p < 0.001.*

**Table S10.** Output from full model on beliefs of disease transmission (Multinomial Mixed Model – MXL Model). “No secondary education” includes respondents with no education, incomplete or complete fundamental education, and incomplete secondary education; “secondary education” includes respondents with complete secondary education; “more than secondary education” includes respondents with incomplete and complete university degrees and post-graduation studies. “High sighting frequencies” considers daily or weekly sightings of either marmosets or howler monkeys; “medium frequency sightings” considers monthly or occasional sightings from once to four times per year; “low frequency sightings” considers sightings occurring less than once per year, including those who had seen marmosets or howler monkeys only once, never, or did not know/declined to answer. Abbreviations: SE: Standard Error; DK/DA: Do not know/ Do not answer; MXL: Multinomial logit mixed models; GLMM: Generalized Linear Mixed Model.

|  |  | **Fixed effects** | **Estimate** | **SE** | **OR [CI 95%]** | ***p*** |
| --- | --- | --- | --- | --- | --- | --- |
|  | ***1) Do you think that NHPs can transmit diseases?*** | | | | | |
| **MXL model** | “No” versus “Yes” response | | | | | |
|  | Intercept |  | -1.93 | 0.84 | 0.15 [0.03-0.76] | **0.023 *** |
|  | Urbanization | (urban) | 0.48 | 0.39 | 1.62 [0.76-3.44] | 0.211 |
|  | YF-occurrence | (YF-positive) | 0.32 | 0.40 | 1.38 [0.63-3.02] | 0.418 |
|  | Age | (27-39) | 0.19 | 0.62 | 1.21 [0.36-4.07] | 0.754 |
|  |  | (40-52) | 0.36 | 0.57 | 1.43 [0.47-4.39] | 0.534 |
|  |  | (53-64) | -0.20 | 0.66 | 0.82 [0.22-3.01] | 0.767 |
|  |  | (>64) | 0.16 | 0.63 | 1.18 [0.35-4.02] | 0.792 |
|  | Education | (secondary education) | -0.30 | 0.46 | 0.74 [0.30-1.81] | 0.511 |
|  |  | (more than secondary education) | -0.29 | 0.47 | 0.75 [0.30-1.87] | 0.532 |
|  | Gender | (female) | -0.39 | 0.33 | 0.68 [0.35-1.30] | 0.238 |
|  | Sight frequency | (marmoset – medium frequency) | -0.28 | 0.42 | 0.76 [0.33-1.72] | 0.516 |
|  |  | (marmoset – low frequency) | 0.42 | 0.50 | 1.53 [0.56-4.03] | 0.395 |
|  |  | (howler monkey – medium frequency) | -0.89 | 0.73 | 0.41 [0.10-1.72] | 0.223 |
|  |  | (howler monkey – low frequency) | -0.29 | 0.63 | 0.75 [0.22-2.56] | 0.643 |
|  | “DK/DA” versus “Yes” response | | | | | |
|  | Intercept |  | -2.05 | 0.90 | 0.13 [0.02-0.75] | **0.023 *** |
|  | Urbanization | (urban) | -0.51 | 0.29 | 0.60 [0.34-1.06] | **0.077 .** |
|  | YF-occurrence | (YF-positive) | 0.21 | 0.33 | 1.24 [0.65-2.38] | 0.520 |
|  | Age | (27-39) | -0.21 | 0.48 | 0.81 [0.31-2.06] | 0.657 |
|  |  | (40-52) | -0.32 | 0.46 | 0.73 [0.30-1.79] | 0.487 |
|  |  | (53-64) | -0.45 | 0.49 | 0.64 [0.24-1.68] | 0.363 |
|  |  | (>64) | 0.12 | 0.48 | 1.13 [0.44-2.86] | 0.805 |
|  | Education | (secondary education) | 0.38 | 0.32 | 1.46 [0.77-2.75] | 0.246 |
|  |  | (more than secondary education) | -1.02 | 0.51 | 0.36 [0.13-0.97] | **0.045 *** |
|  | Gender | (female) | -0.23 | 0.27 | 0.79 [0.47-1.34] | 0.385 |
|  | Sight frequency | (marmoset – medium frequency) | 0.19 | 0.31 | 1.21 [0.66-2.22] | 0.543 |
|  |  | (marmoset – low frequency) | 0.63 | 0.41 | 1.87 [0.84-4.17] | 0.123 |
|  |  | (howler monkey – medium frequency) | 0.68 | 0.82 | 1.98 [0.39-9.93] | 0.408 |
|  |  | (howler monkey – low frequency) | 1.09 | 0.79 | 2.99 [0.64-14.03] | 0.166 |

|  |  | **Fixed effects** | **Estimate** | **SE** | **OR [CI 95%]** | ***p*** |
| --- | --- | --- | --- | --- | --- | --- |
|  | ***2) Are you afraid that NHPs can transmit diseases to you?*** | | | | | |
| **MXL model** | “No” versus “Yes” response | | | | | |
|  | Intercept |  | 0.10 | 0.35 | 1.11 [0.56-2.22] | 0.769 |
|  | Urbanization | (urban) | 0.08 | 0.22 | 1.08 [0.71-1.66] | 0.707 |
|  | YF-occurrence | (YF-positive) | -0.07 | 0.22 | 0.94 [0.60-1.45] | 0.763 |
|  | Age | (27-39) | -0.43 | 0.36 | 0.65 [0.32-1.32] | 0.235 |
|  |  | (40-52) | -0.60 | 0.34 | 0.55 [0.28-1.08] | **0.082 .** |
|  |  | (53-64) | -0.67 | 0.38 | 0.51 [0.25-1.08] | **0.077 .** |
|  |  | (>64) | -0.22 | 0.37 | 0.80 [0.39-1.67] | 0.556 |
|  | Education | (secondary education) | 0.32 | 0.26 | 1.38 [0.82-2.31] | 0.229 |
|  |  | (more than secondary education) | 0.59 | 0.30 | 1.81 [1.01-3.23] | **0.045 *** |
|  | Gender | (female) | -0.67 | 0.20 | 0.51 [0.35-0.76] | **0.001 ***** |
|  | “DK/DA” versus “Yes” response | | | | | |
|  | Intercept |  | -1.67 | 0.84 | 0.19 [0.04-0.98] | **0.048*** |
|  | Urbanization | (urban) | -1.01 | 0.66 | 0.37 [0.10-1.34] | 0.129 |
|  | YF-occurrence | (YF-positive) | -0.26 | 0.69 | 0.77 [0.20-2.98] | 0.707 |
|  | Age | (27-39) | -1.21 | 0.97 | 0.30 [0.05-1.97] | 0.208 |
|  |  | (40-52) | -1.74 | 0.97 | 0.18 [0.03-1.18] | 0.073 |
|  |  | (53-64) | -0.72 | 0.86 | 0.49 [0.09-2.62] | 0.400 |
|  |  | (>64) | -1.10 | 1.00 | 0.33 [0.05-2.35] | 0.270 |
|  | Education | (secondary education) | -0.03 | 0.86 | 0.97 [0.18-5.25] | 0.975 |
|  |  | (more than secondary education) | 0.83 | 0.84 | 2.30 [0.45-11.85] | 0.318 |
|  | Gender | (female) | 0.01 | 0.59 | 1.01 [0.32-3.21] | 0.981 |
| ____________________________________________________________________________________ | | | | | | |
| **GLMM** | Intercept |  | -0.11 | 0.20 | 0.89 [0.60-1.33] | 0.584 |
|  | Gender | (female) | -0.61 | 0.20 | 0.55 [0.37-0.81] | **0.002**** |

**Table S11.** Output from the models on fear of disease transmission: i) Multinomial Mixed Model (MXL Model) with all predictors; ii) Generalized Linear Mixed Model (GLMM) with AIC-selected predictors. “No secondary education” includes respondents with no education, incomplete or complete fundamental education, and incomplete secondary education; “secondary education” includes respondents with complete secondary education; “more than secondary education” includes respondents with incomplete and complete university degrees and post-graduation studies. “High sighting frequencies” considers daily or weekly sightings of either marmosets or howler monkeys; “medium frequency sightings” considers monthly or occasional sightings from once to four times per year; “low frequency sightings” considers sightings occurring less than once per year, including those who had seen marmosets or howler monkeys only once, never, or did not know/declined to answer. Abbreviations: SE: Standard Error; DK/DA: Do not know/ Do not answer; MXL: Multinomial logit mixed models; GLMM: Generalized Linear Mixed Model.

| **Table S12.** Variance Inflation Factors (VIFs) for fixed-effect predictors included in the perception and disease-risk models. | | | | |
| --- | --- | --- | --- | --- |
| **Predictors** | **Marmoset perception** | **Howler monkey perception** | **Disease belief model** | **Fear model** |
| YF-status | 1.16 | 1.22 | 1.67 | 1.40 |
| Urbanization | 1.09 | 1.12 | 2.06 | 1.72 |
| Age group | 1.03 | 1.02 | 1.38 | 1.34 |
| Education level | 1.09 | 1.09 | 1.25 | 1.26 |
| Gender | 1.03 | 1.04 | 1.44 | 1.40 |
| Marmoset sighting frequency | 1.04 | - | 1.18 | - |
| Howler monkey sighting frequency | - | 1.08 | 2.03 | - |
| * Values correspond to adjusted GVIFs (GVIF^(1/2 x Df)) to account for predictors with multiple degrees of freedom. Values <3 indicate low multicollinearity among predictors | | | | |
