# Supplementary material for: Yellow Fever Outbreaks Are Not Associated With Long‐Term Negative Human Perceptions of Wild Primates in Brazil
Source: Am J Primatol. 2026 Oct 21;88(10):e70219. doi: 10.1002/ajp.70219 (PMC13685054; doi:10.1002/ajp.70219)
Supplement: Supplementary file 2 [file AJP-88-e70219-s002.docx]

**Supporting Information S2:**

**Yellow fever outbreaks are not associated with long-term negative human perceptions of wild primates in Brazil**

**Semi-structured interview in portuguese**

V2.Percepção Humana Dos Primatas Não Humanos No

Brasil

Nome pesquisador

ID

*Cada pesquisador começa com o número 1 e segue sua própria contagem. Cada pesquisador deve saber a qual número de pesquisa está atribuído. É necessário anotá-lo no formulário de consentimento informado (número e nome do pesquisador)..*

Município (+/- comunidad)

Escolha a opção adequada para o local do questionário atual:

 Urbano + Febre Amarela positivo Urbano + Febre Amarela negativo Rural + Febre Amarela positivo Rural + Febre Amarela negativo

Data

yyyy-mm-dd

# A. Conhecimento e contato com macacos

1. Você ja viu macacos na região?

 Sim Não NS/NR

2.1. Você pode me dizer qual destes é o sagui? (Pode ser mais de uma foto)

*Usar nome da região: Sagui, Sagüim, Sauim, Xauim, Sauí, Soin, Massau, Tamari, Mico, Nico, Miquinho*

 1 2 3 4 5 6 7 8 9 10 11

12 NS/NR

2.2. Você pode me dizer qual destes é o bugio?

*Usar nome da região: Bugio, Barbado, Guariba*

 1 2 3 4 5 6 7 8 9 10 11

12 NS/NR

3.1. Com que frequência você tem avistado ***Callithrix***?

*Usar nome da região: Sagui, Sagüim, Sauim, Xauim, Sauí, Soin, Massau, Tamari, Mico, Nico, Miquinho*

 Todos os dias Toda semana 1 vez/mês

 1 vez/ano Outro Nunca NS/NR

3.1. Especificar outro

*Da pergunta: 3.1. Com que freqüência você tem avistado Callithrix?*

3.2. Com que frequência você tem avistado ***Alouatta***?

*Usar nome da região: Bugio, Barbado, Guariba*

 Todos os dias Toda semana 1 vez/mês

 1 vez/ano Outro Nunca NS/NR

3.2. Especificar outro

*Da pergunta: 3.3. Com que freqüência você tem avistado Alouatta?*

3.3. Você tem avistado macacos de outra/s espécie/s?

*Insira as espécies somente se você quiser adicioná-las. Caso contrário, ignore a pergunta.*

 Macaco-prego (Sapajus) Guigó (Callicebus)

 Coatá ou Macaco aranha (Ateles) Mico-leão (Leontopithecus)

 Muriqui (Brachyteles) Outros (especificar) Não viram

3.3. Especificar outro

*Da pergunta: 3.3. Você tem avistado primatas de outra espécie?*

3.3.1. Com que frequência você tem avistado macacos das espécies que você falou?

*Escreva uma frequência para cada espécie mencionada.*

4.1. Em quais meses do ano você vê a ***Callithrix*** com mais frequência?

*Usar nome da região: Sagui, Sagüim, Sauim, Xauim, Sauí, Soin, Massau, Tamari, Mico, Nico, Miquinho*

| Janeiro | Fevereiro  | Março  | Abril  | Maio | Junho  | Julho  |
| --- | --- | --- | --- | --- | --- | --- |
| Agosto  NS/NR | Setembro  | Outubro  |  Novembro |  | Dezembro | Não viram  |

4.2. Em quais meses do ano você vê a ***Alouatta*** com mais frequência?

*Usar nome da região: Bugio, Barbado, Guariba*

| Janeiro | Fevereiro  | Março  | Abril  | Maio | Junho  | Julho  |
| --- | --- | --- | --- | --- | --- | --- |
| Agosto  NS/NR | Setembro  | Outubro  |  Novembro |  | Dezembro | Não viram  |

5.1. Onde você observou a os ***Callithrix*** com mais frequência durante o último ano?

*Usar nome da região: Sagui, Sagüim, Sauim, Xauim, Sauí, Soin, Massau, Tamari, Mico, Nico, Miquinho*

 Não os viram (resposta única) Árvores Perto do rio

 Praia Chão Perto de água parada

No jardim de minha casa	Dentro de minha casa	Casa de um parente ou amigo

 Jardim público

5.1. Especificar outro

Outro	NS/NR (resposta única)

*Da pergunta: 5.1. Onde você observou a os Callithrix com mais freqüência durante o último ano?*

5.2. Onde você observou a os ***Alouatta*** com mais frequência durante o último ano?

*Usar nome da região: Bugio, Barbado, Guariba*

 Não os viram (resposta única) Árvores Perto do rio

 Praia Chão Perto de água parada

No jardim de minha casa	Dentro de minha casa	Casa de um parente ou amigo

Jardim público

5.2. Especificar outro

Outro	NS/NR (resposta única)

*Da pergunta: 5.3. Onde você observou a os Alouatta com mais freqüência durante o último ano?*

6.1. Você já interagiu de alguma forma com a ***Callithrix***?

*Usar nome da região: Sagui, Sagüim, Sauim, Xauim, Sauí, Soin, Massau, Tamari, Mico, Nico, Miquinho*

Sim	Não	NS/NR

6.1.1. Você poderia me dizer como aconteceu este contato?

*Usar nome da região: Sagui, Sagüim, Sauim, Xauim, Sauí, Soin, Massau, Tamari, Mico, Nico, Miquinho*

| Toquei no primata | É meu animal de estimação | Eu dei comida para ele |
| --- | --- | --- |
| Roubou comida de mim | Entrou no minha casa | Me mordeu |
| Entrou em meu trabalho  NS/NR | Defecou perto de mim | Outro |

6.1.1. Especificar outro

*Da pergunta: 6.1.1. Você poderia me dizer como aconteceu este contato (Callithrix)?*

6.2. Você já interagiu de alguma forma com a ***Alouatta***?

*Usar nome da região: Bugio, Barbado, Guariba*

Sim	Não	NS/NR

6.2.1. Você poderia me dizer como aconteceu este contato?

*Usar nome da região: Bugio, Barbado, Guariba*

| Toquei no primata | É meu animal de estimação | Eu dei comida para ele |
| --- | --- | --- |
| Roubou comida de mim | Entrou no minha casa | Me mordeu |
| Entrou em meu trabalho NS/NR | Defecou perto de mim | Outro |
| 6.2.1. Especificar outro |  |  |

*Da pergunta: 6.2.1. Você poderia me dizer como aconteceu este contato (Alouatta)?*

6.3. Você já interagiu de alguma forma com a/s outra/s espécie/s de macaco/s que você falou no início?

*Pergunta sobre espécies mencionadas que não sejam Callithrix e Alouatta*

Sim Não NS/NR

6.3.1. Você poderia me dizer como aconteceu este contato?

Toquei no primata	É meu animal de estimação	Eu dei comida para ele Roubou comida de mim	Entrou no minha casa	Me mordeu

Entrou em meu trabalho NS/NR

6.3.1. Especificar outro

Defecou perto de mim	Outro

*Da pergunta: 6.3.1. Você poderia me dizer como aconteceu este contato?*

7.1. Alguma vez você já alimentou a ***Callithrix***?

*Usar nome da região: Sagui, Sagüim, Sauim, Xauim, Sauí, Soin, Massau, Tamari, Mico, Nico, Miquinho*

Sim	Não	NS/NR

7.1.1. Com que freqüência você alimenta a ***Callithrix***?

*Usar nome da região: Sagui, Sagüim, Sauim, Xauim, Sauí, Soin, Massau, Tamari, Mico, Nico, Miquinho*

Todos os dias	Todas as semanas	1 vez/mês

1 vez/ano	NS/NR

7.1.1.1. Você usa um local específico para a estação de alimentação (feeding-station)?

Sim	Não	NS/NR

7.2. Alguma vez você já alimentou a ***Alouatta***?

*Usar nome da região: Bugio, Barbado, Guariba*

Sim	Não	NS/NR

7.2.1. Com que freqüência você alimenta a ***Alouatta***?

*Usar nome da região: Bugio, Barbado, Guariba*

Todos os dias	Todas as semanas	1 vez/mês

1 vez/ano	NS/NR

7.2.1.1. Você usa um local específico para a estação de alimentação (feeding-station)?

Sim	Não	NS/NR

7.3. Alguma vez você já alimentou a/s outra/s espécie/s de macaco/s que você falou no início?

*Pergunta sobre espécies mencionadas que não sejam Callithrix e Alouatta*

Sim	Não	NS/NR

7.3.1. Com que freqüência você alimenta a essas espécies?

*Pergunta sobre espécies mencionadas que não sejam Callithrix e Alouatta*

Todos os dias	Todas as semanas	1 vez/mês

1 vez/ano	NS/NR

7.3.1.1. Você usa um local específico para a estação de alimentação (feeding-station)?

Sim	Não	NS/NR

8.1. Você ja foi mordido por um macaco?

Sim Não

NS/NR

8.1.1. Quais espécie/s de macaco/s morderam você?

Sagui, Soin, Mico, Nico (Callithrix) Bugio, Guariba, Barbado (Alouatta) Macaco-prego (Sapajus)

Outro (especificar) NS/NR

8.1.1. Especificar outro

*Da pergunta: 8.1.1. Quais espécie/s de macaco/s morderam você?*

8.1.2. Há quanto tempo foi?

*Especificar para cada espécie*

8.1.3. Após ter sido mordido, que ação você tomou?

Não fiz nada

Lavei só com água

Lavei com água e sabão Fui ao posto de saúde

Fui ao posto de saúde e me deram uma vacina Outro

NS/NR

8.1.3. Especificar outro

*Da pergunta: 8.1.3. Após ter sido mordido, que ação você tomou?*

9. Você ouve macacos vocalizando?

Sim	Não	NS/NR

9.1. Que espécies de macacos você ouve vocalizando?

Sagui, Soin, Mico, Nico (Callithrix)

Bugio, Guariba, Barbado (Alouatta)

Macaco-prego (Sapajus)

Outro	NS/NR

9.1. Especificar outro:

*Da pergunta: 9.1. Qué espécie de macacos você ouve vocalizando?*

9.1.1. Com que frequência você ouve ***Callithrix***?

*Usar nome da região: Sagui, Sagüim, Sauim, Xauim, Sauí, Soin, Massau, Tamari, Mico, Nico, Miquinho*

Todos os dias	Todas as semanas	1 vez/mês

1 vez/ano	< 1 vez/ano	Outro NS/NR

9.1.1. Especificar outro:

*Da pergunta: 9.1.1. Com que frequência você ouve Callithrix?*

9.1.2. Com que frequência você ouve ***Alouatta***?

*Usar nome da região: Bugio, Barbado, Guariba*

Todos os dias	Todas as semanas	1 vez/mês

1 vez/ano	< 1 vez/ano	Outro NS/NR

9.1.2. Especificar outro:

*Da pergunta: 9.1.2. Com que frequência você ouve Alouatta?*

10. Você sabe se há macacos como animais de estimação (pets) na região?

*Se alguém que você conhece ou sabe que tem macacos como animal de estimação*

Sim	Não	NS/NR

10.1. Quais espécies?

Sagui, Soin, Mico, Nico (Callithrix)

Bugio, Guariba, Barbado (Alouatta)

Macaco-prego (Sapajus)

Outro	NS/NR

10.1. Especificar outro

*Da pergunta: 10.1. Quais outras espécies?*

11. Você já viu macacos próximos de animais domésticos?

Sim	Não	NS/NR

11.1. Quais espécies?

Sagui, Soin, Mico, Nico (Callithrix)

Bugio, Guariba, Barbado (Alouatta)

Macaco-prego (Sapajus)

Outro	NS/NR

11.1. Especificar outro

*Da pergunta: 11.1 Quais espécies ja viu proximas de animais domésticos?*

11.1.1. Com que freqüência ja viu ***Callithrix*** próximo de animais domésticos?

*Usar nome da região: Sagui, Sagüim, Sauim, Xauim, Sauí, Soin, Massau, Tamari, Mico, Nico, Miquinho*

1 vez	1 vez/mês	< 1 vez/mês

Cada 2 semanas	Todas as semanas	Todos os dias

Outro	NS/NR

11.1.1 Especificar outro:

*Da pergunta: 11.1.1. Com que freqüência ja viu Callithrix próximo de animais domésticos?*

11.1.2. Com que freqüência ja viu ***Alouatta*** próximo de animais domésticos?

*Usar nome da região: Bugio, Barbado, Guariba*

1 vez

1 vez/mês

< 1 vez/mês

Cada 2 semanas Todas as semanas Todos os dias

Outro NS/NR

11.1.2. Especificar outro:

*Da pergunta: 11.1.2. Com que freqüência ja viu Alouatta próximo de animais domésticos?*

11.2. Com que animal doméstico?

Gato	Cachorro	Outro NS/NR

11.2. Especificar outro

*Da pergunta: 11.2. Com que animal doméstico?*

11.3. Que tipo de interação eles tiveram?

Brincadeira / Atitude de jogo	Eles mordem	Vocalização sem tocar

O animal de estimação incomodou o macaco

NS/NR

11.3. Especificar outro

O macaco incomodou a animal de estimação

Outro

*Da pergunta: 11.3. Que tipo de interação eles tiveram?*

12. Você já viu macacos se alimentar de fontes de alimentos humanos?

*Considera-se alimento humano aquele que se destina a ser consumido por seres humanos, podendo ser alimentos cozidos, alimentos processados, mas também frutas e vegetais destinados ao consumo humano, mais não alimentos silvestres..*

Sim	Não	NS/NR

12.1. Quais espécies?

Sagui, Soin, Mico, Nico (Callithrix)

Bugio, Guariba, Barbado (Alouatta)

Macaco-prego (Sapajus)

Outro	NS/NR

12.1. Especificar outro

*Da pergunta: 12.1. Quais outras espécies viu alimentar de fontes de alimento humano?*

12.2.1. ***Callithrix***: De que fonte?

*Usar nome da região: Sagui, Sagüim, Sauim, Xauim, Sauí, Soin, Massau, Tamari, Mico, Nico, Miquinho*

Alguém o alimentou	Lixo	Roubou comida sem vigilância

Minhas árvores frutíferas/ minha colheita

Outro

12.2.1. Especificar outro

*Da pergunta: 12.2.1. Callithrix: De que fonte?*

12.2.2. ***Alouatta***: De que fonte?

*Usar nome da região: Bugio, Barbado, Guariba*

Alguém o alimentou	Lixo	Roubou comida sem vigilância

Minhas árvores frutíferas/ minha colheita

Outro

12.2.2. Especificar outro

*Da pergunta: 12.2.2. Alouatta: De que fonte?*

13. Você conhece alguma lenda, crença social ou superstição sobre alguma espécie de macaco?

Sim Não NS/NR

13.1. Quais espécies?

Sagui, Soin, Mico, Nico (Callithrix)

Bugio, Guariba, Barbado (Alouatta)

Macaco-prego (Sapajus)

Outro	NS/NR

13.1. Especifique outro:

*Da pergunta: 13. Você conhece alguma lenda, crença social ou superstição sobre alguma espécie de macaco?*

13. Explique brevemente a lenda, superstição ou crença.

*Importante: especifique tambén a espécie de macaco*

14. Você já presenciou comportamentos negativos de pessoas em relação aos macacos?

Sim Não NS/NR

14.1. Quais espécies?

Sagui, Soin, Mico, Nico (Callithrix)

Bugio, Guariba, Barbado (Alouatta)

Macaco-prego (Sapajus)

Outro	NS/NR

14. Especificar outro

*Da pergunta: 14. Você já presenciou comportamentos negativos de pessoas em relação às macacos?*

14.1. Que tipo de comportamento?

FIM PARTE (A). Observações/comentários:

*Somente se necessário, caso contrário, passe para a próxima pergunta.*

# B. Percepção das espécies

15. Se você pudesse escolher, a quantidade de saguis nesta região…

*Callithrix (Sagui, Sagüim, Sauim, Xauim, Sauí, Soin, Massau, Tamari, Mico, Nico, Miquinho)*

Diminuiria muito	Diminuiria	Seria mantido

Aumentaria	Aumentaria muito	Sem opinão

16. Se os saguis desaparecessem da área, você ficaria…

*Callithrix (Sagui, Sagüim, Sauim, Xauim, Sauí, Soin, Massau, Tamari, Mico, Nico, Miquinho)*

Muito feliz	Feliz	Indiferente

Triste	Muito triste	Sem opinão

17. O que você sente quando vê um sagui?

*Callithrix (Sagui, Sagüim, Sauim, Xauim, Sauí, Soin, Massau, Tamari, Mico, Nico, Miquinho)*

Odeio	Não gosto	Indiferente

Eu gosto	Gosto muito	Sem opinão

18. Os saguis são…

*Callithrix (Sagui, Sagüim, Sauim, Xauim, Sauí, Soin, Massau, Tamari, Mico, Nico, Miquinho)*

Um grande problema	Um problema	Indiferente

Um benefício	Um grande benefício	Sem opinão

19. Minhas experiências com saguis são…

*Callithrix (Sagui, Sagüim, Sauim, Xauim, Sauí, Soin, Massau, Tamari, Mico, Nico, Miquinho)*

Sempre negativo	Geralmente negativo	Indiferente

Geralmente positivo	Sempre positivo	Sem opinão

20. Acredito que os saguis têm um efeito sobre a fauna selvagem e o meio ambiente natural...

*Callithrix (Sagui, Sagüim, Sauim, Xauim, Sauí, Soin, Massau, Tamari, Mico, Nico, Miquinho)*

Muito prejudicial	Prejudicial	Não tem efeito

Benéfico	Muito benéfico	Sem opinão

21. Se você pudesse escolher, a quantidade de bugios nesta região…

*Alouatta (Bugio, Barbado, Guariba)*

Diminuiria muito	Diminuiria	Seria mantido

Aumentaria	Aumentaria muito	Sem opinão

22. Se os bugios desaparecessem da área, você ficaria…

*Alouatta (Bugio, Barbado, Guariba)*

Muito feliz	Feliz	Indiferente

Triste	Muito triste	Sem opinão

23. O que você sente quando vê um bugio?

*Alouatta (Bugio, Barbado, Guariba)*

Odeio	Não gosto	Indiferente

Eu gosto	Gosto muito	Sem opinão

24. Os bugios são…

*Alouatta (Bugio, Barbado, Guariba)*

| Um grande problema | Um problema | Indiferente |
| --- | --- | --- |
| Um benefício | Um grande benefício | Sem opinão |
| 25. Minhas experiências com bugios são… | | |
| *Alouatta (Bugio, Barbado, Guariba)* |  |  |
| Sempre negativo | Geralmente negativo | Indiferente |
| Geralmente positivo | Sempre positivo | Sem opinão |

26. Acredito que os bugios têm um efeito sobre a fauna selvagem e o meio ambiente natural...

*Alouatta (Bugio, Barbado, Guariba)*

Muito prejudicial	Prejudicial	Não tem efeito

Benéfico	Muito benéfico	Sem opinão

OPCIONAL: * Percepção de outras espécies de primatas (especificar a espécie)

*Opcional*

27. Se você pudesse escolher, a abundância de	nesta região…

Diminuiria muito	Diminuiria	Seria mantido

Aumentaria	Aumentaria muito	Sem opinão

28. Se os macacos	desaparecessem da área, você se sentiria…

Muito feliz	Feliz	Indiferente

Triste	Muito triste	Sem opinão

29. O que você sente quando vê um macaco	?

Odeio	Não gosto	Indiferente

Eu gosto	Gosto muito	Sem opinão

| 30. Os macacos são… |  | |
| --- | --- | --- |
| Um grande problema | Um problema | Indiferente |
| Um benefício | Um grande benefício | Sem opinão |

31. Minhas experiências com	são…

Sempre negativo	Geralmente negativo	Indiferente

Geralmente positivo	Sempre positivo	Sem opinão

32. Acredito que os macacos _ _ _ _ _ _ _ _ têm um efeito sobre a fauna selvagem e o meio ambiente natural...

Muito prejudicial	Prejudicial	Não tem efeito

Benéfico	Muito benéfico	Sem opinão

FIM PARTE (B). Observações/comentários:

*Somente se necessário, caso contrário, passe para a próxima pergunta. Adicione comentários importantes mencionados pelos entrevistados durante as perguntas.*

# C. Aceitabilidade das estratégias de redução de contato

33. Quando humanos alimentam primatas, há...

*Selecione a opção mais adequada para você*  Um benefício para o primata Um benefício para o humano Um benefício para ambos

Um dano a ambos

Danos aos primatas Danos aos humanos NS/NR

34. Acredito que o contato de convivência entre humanos e macacos deve…

*Selecione a opção mais adequada para você*

Aumentar a frequência Diminuir a frequência Fique como está

NS/NR

35. O que você acha de fazer control para a redução da população de macacos? (criar estratégias para reduzir o número de macacos)

*Selecione a opção mais adequada para você*

Discordo fortemente Não concordo

Neutro Concordo

Totalmente de acordo Sem opinão

36. O que você acha de mudar os macacos para outras áreas que não as atuais? (translocação)

*Selecione a opção mais adequada para você*

Discordo fortemente Não concordo

Neutro Concordo

Totalmente de acordo Sem opinão

37. O que você acha de proibir e multar pessoas que alimentam macacos?

*Selecione a opção mais adequada para você*

Discordo fortemente Não concordo

Neutro Concordo

Totalmente de acordo Sem opinão

38. O que você acha de colocar redes de proteção em edifícios / casas / plantaçoes vulneráveis à entrada de macacos?

*Selecione a opção mais adequada para você*

Discordo fortemente Não concordo

Neutro Concordo

Totalmente de acordo Sem opinão

39. O que você acha do governo local fornecer alimentos adequados para incentivar os macacos a permanecerem em áreas verdes adequadas para eles

*Selecione a opção mais adequada para você*

Discordo fortemente Não concordo

Neutro Concordo

Totalmente de acordo Sem opinão

OPCIONAL: Você tem alguma ideia ou sugestão de intervenção em populações de macacos?

*Somente se necessário, caso contrário, passe para a próxima pergunta.*

FIM PARTE (C). Observações/comentários:

*Somente se necessário, caso contrário, passe para a próxima pergunta.*

# D. Percepção de risco

40. Você acha que os macacos podem transmitir doenças as pessoas?

Sim Não NS/NR

40.1. Quais espécies:

Sagui, Soin, Mico, Nico (Callithrix) Bugio, Guariba, Barbado (Alouatta) Macaco-prego (Sapajus)

Todos os macacos Outro (especificar) NS/NR

40.1. Especificar outro:

*Da pergunta: 40.1. Quais especies*

41. Você tem medo que os macacos transmitam alguma doença para você?

Sim Não

NS/NR

41.1. Quais espécies:

Sagui, Soin, Mico, Nico (Callithrix) Bugio, Guariba, Barbado (Alouatta) Macaco-prego (Sapajus)

Todos os macacos Outro (especificar) NS/NR

41.1. Especificar outro

*Da pergunta: 41.1. Quais espécies:*

42. Você acha que a raiva pode ser transmitida por macacos?

Sim Não NS/NR

42.1. Quais espécies:

Sagui, Soin, Mico, Nico (Callithrix) Bugio, Guariba, Barbado (Alouatta) Macaco-prego (Sapajus)

Todos os macacos Outro (especificar) NS/NR

42.1. Especificar outro

*Da pergunta: 42.1. Quais espécies:*

43. Você acha que os macacos podem ser infectados pela COVID-19?

Sim Não NS/NR

44. Você acha que os macacos podem infectar humanos com COVID-19?

Sim Não

NS/NR

45. Você acha que a febre amarela pode ser transmitida aos humanos por macacos?

Sim Não NS/NR

45.1. Quais espécies:

Sagui, Soin, Mico, Nico (Callithrix) Bugio, Guariba, Barbado (Alouatta) Macaco-prego (Sapajus)

Todos os macacos Outro (especificar) NS/NR

45.1. Especificar outro

*Da pergunta: 45.1. Quais espécies:*

46. Você acha que a varíola do macaco pode ser transmitda aos humanos por macacos?

Sim Não NS/NR

46.1. Quais espécies:

Sagui, Soin, Mico, Nico (Callithrix) Bugio, Guariba, Barbado (Alouatta) Macaco-prego (Sapajus)

Todos os macacos Outro (especificar) NS/NR

46.1. Especificar outro

*Da pergunta: 46.1. Quais espécies:*

47. Você observou algum macaco doente?

Sim Não NS/NR

47.1. Quando?
47.2. Quais espécies?

Sagui, Soin, Mico, Nico (Callithrix) Bugio, Guariba, Barbado (Alouatta) Macaco-prego (Sapajus)

Outro (especificar NS/NR

47.2. Especificar outro

*Da pergunta: 47.2. Quais outras espécies observou doentes?*

47.3. O que você fez?

Não fiz nada

Levei-o para casa

Dei comida e/ou água para ele Informei a polícia

Avisado ou levado ao veterinário

Notifiquei o serviço de saúde ou liguei para o Disque Saúde (136) Outros

47.3. Especificar outro

*Da pergunta: 47.3. O que você fez?*

48. Você já se deparou com um macaco morto?

Sim Não NS/NR

48.1. Quando?
48.2. Quais espécies?

Sagui, Soin, Mico, Nico (Callithrix) Bugio, Guariba, Barbado (Alouatta) Macaco-prego (Sapajus)

Outro (especificar) NS/NR

48.3. O que você fez?

Não fiz nada

Deixei-o onde estava Eu enterrei

Informei a polícia Avisei ao veterinário

Notifiquei o serviço de saúde ou liguei para o Disque Saúde (136) Outros

48.3. Especificar outro:

49. Alguém da família ou morador dessa casa, já teve alguma doença como:

Não Dengue

Toxoplasmose Malaria

Zika Raiva

Chikungunya

Calazar (leishmaniose) Febre amarela

Doença de Chagas Verminose

Diarrea por vermes Sarna/escabiose Outro

49.1. Especificar outro

*Da pergunta: 49. Alguém da família ou morador dessa casa, já teve alguma doença como:*

49.1. À quanto tempo?

50. Conhece alguém da região que pegou?

Não Dengue

Toxoplasmose Malaria

Zika Raiva

Chikungunya

Calazar (leishmaniose) Febre amarela

Doença de Chagas Verminose

Diarrea por vermes Sarna/escabiose Outro

50. Especificar outro

*Da pergunta: 50. Conhece alguém da região que pegou?*

50.1. À quanto tempo?

51. No local onde observa macacos, há muriçocas / pernilongas (mosquitos)?

Sim Não NS/NC

52. Perturbam muito os muriçocas / pernilongas (mosquitos)?

Sim Não NS/NC

53. O ano todo?

Sim Não

NS/NC

54. Qual época do ano?

Chuva Seca

Meses (somente se eles especificarem apenas alguns meses além dessas estações) NS/NR

54.1. Meses:

Janeiro Fevereiro Março Abril

Maio Junho Julho Agosto

Setembro Outubro Novembro Dezembro Não viram NS/NR

55. Que horário costumam picar?

Manhã Tarde Noite NS/NR

FIM PARTE (D). Observações/comentários:

*Somente se necessário, caso contrário, passe para a próxima pergunta.*

# E. Identificação

Nome:

Contato:

Sexo:

Feminino Masculino Outro

NS/NR

Faixa etária (anos):

Escolaridade:

Não tenho

Ensino fundamental incompleto Ensino fundamental completo Ensino médio incompleto

Ensino médio completo

Ensino superior incompleto (universitário, técnico) Ensino superior completo (universitário, técnico)

Estudos de pós-graduação (mestrado, doutorado, especialidade médica) NS/NR

Localização GPS automática (de prefêrencia)

latitude (x.y °)

longitude (x.y °)

altitude (m)

accuracy (m)

Localizaçao GPS manual (só se a anterior não tiver funcionado)

*Introduzir a referência geográfica (latitude e longitude utilizando o Google Maps ou, na sua falta, o nome do local específico).*

**Figure S1**. Images of different wildlife species used during the interview to identify marmosets (numbers 8 and 12) and howler monkeys (numbers 3 and 4).

1 - Chimpanzee, *Pan troglodytes* (Abeselom Zerit, n.d., Shutterstock); 2- Bald uakari, *Cacajao calvus* (Scottisom, 2012, iNaturalist); 3- Black and gold howler monkey, *Alouatta caraya* (Juniors Bildarchiv, n.d.); 4- Brown howler monkey*, Alouatta guariba* (Peter Schoen, 2010, Flickr); 5- Three-fingered sloth, *Bradypus variegatus* (unknown; n.d., iNaturalist); 6- Ring-tailed lemur, *Lemur catta* (Charles Cantin, n.d.); 7- Tufted capuchin, *Sapajus apella* (Arthur Chapman, 2012, Flickr); 8- Common marmoset, *Callithrix jacchus* (Gabriel Paladino, n.d., Flickr); 9- Wooly spider monkey, *Brachyteles arachnoides* (Leonardo Desordi Lobo, 2020, Flickr); 10- Big-eared opossum, *Didelphis aurita* (n.d., Lifecatalog); 11- Orangutan, *Pongo* sp. (Mariana Alva, 2012, Flickr); 12- Black-tufted marmoset, *Callithrix penicillata* (Esteban Argerich, n.d., Fotonat).
